# Brain Anatomical Network and Intelligence

**DOI:** 10.1371/journal.pcbi.1000395

**Published:** 2009-05-29

**Authors:** Yonghui Li, Yong Liu, Jun Li, Wen Qin, Kuncheng Li, Chunshui Yu, Tianzi Jiang

**Affiliations:** 1 LIAMA Center for Computational Medicine, National Laboratory of Pattern Recognition, Institute of Automation, Chinese Academy of Sciences, Beijing, China; 2 National Key Laboratory of Cognitive Neuroscience and Learning, Beijing Normal University, Beijing, China; 3 Department of Radiology, Xuanwu Hospital of Capital Medical University, Beijing, China; Indiana University, United States of America

## Abstract

Intuitively, higher intelligence might be assumed to correspond to more efficient information transfer in the brain, but no direct evidence has been reported from the perspective of brain networks. In this study, we performed extensive analyses to test the hypothesis that individual differences in intelligence are associated with brain structural organization, and in particular that higher scores on intelligence tests are related to greater global efficiency of the brain anatomical network. We constructed binary and weighted brain anatomical networks in each of 79 healthy young adults utilizing diffusion tensor tractography and calculated topological properties of the networks using a graph theoretical method. Based on their IQ test scores, all subjects were divided into general and high intelligence groups and significantly higher global efficiencies were found in the networks of the latter group. Moreover, we showed significant correlations between IQ scores and network properties across all subjects while controlling for age and gender. Specifically, higher intelligence scores corresponded to a shorter characteristic path length and a higher global efficiency of the networks, indicating a more efficient parallel information transfer in the brain. The results were consistently observed not only in the binary but also in the weighted networks, which together provide convergent evidence for our hypothesis. Our findings suggest that the efficiency of brain structural organization may be an important biological basis for intelligence.

## Author Summary

Networks of interconnected brain regions coordinate brain activities. Information is processed in the grey matter (cortex and subcortical structures) and passed along the network via whitish, fatty-coated fiber bundles, the white matter. Using maps of these white matter tracks, we provided evidence that higher intelligence may result from more efficient information transfer. Specifically, we hypothesized that higher IQ derives from higher global efficiency of the brain anatomical network. Seventy-nine healthy young adults were divided into general and high IQ groups. We used diffusion tensor tractography, which maps brain white matter fibers, to construct anatomical brain networks for each subject and calculated the network properties using both binary and weighted networks. We consistently found that the high intelligence group's brain network was significantly more efficient than was the general intelligence group's. Moreover, IQ scores were significantly correlated with network properties, such as shorter path lengths and higher overall efficiency, indicating that the information transfer in the brain was more efficient. These converging evidences support the hypothesis that the efficiency of the organization of the brain structure may be an important biological basis for intelligence.

## Introduction

Researchers have long studied the biological basis for intelligence and have found increasing evidence relating high performance on intelligence quotient (IQ) tests to the coordination of multiple brain regions, utilizing both structural and functional brain imaging techniques [1]–[11]. Our hypothesis, inspired by these earlier findings, is that higher IQ test scores may correspond to more efficient information transfer in the brain. However, no direct evidence has been provided from the perspective of brain networks. In particular the relationship between individual intelligence and topological properties of the brain anatomical network has never been investigated, leaving the impact of brain structural organization on intelligence largely unknown.

It is well accepted that the human brain, which can be viewed as a large, interacting and complex network with nontrivial topological properties [12]–[17], especially with small-world attributes, characterized by a high clustering index and a short average distance between any two nodes [18], is one of the most challenging systems found in nature. Noninvasive investigation of human brain networks has been enabled by recent advances in modern neuroimaging techniques. Small-world attributes have been found in brain functional networks using electroencephalography, magnetoencephalography and functional magnetic resonance imaging [13]–[17],[19]. Also, recent progress has been made in the investigation of brain anatomical networks by He et al. [20], who investigated patterns of anatomical connections in cerebral cortices *in vivo* using cortical thickness measured from structural magnetic resonance imaging (MRI). Their findings supported the view that human brain anatomical networks manifest small-world attributes. However, only one binary anatomical network could be generated from a group of subjects by their method, which made it inapplicable for investigating the network properties of an individual brain. In addition to He et al.'s cortical thickness measurements, an anatomical network was derived from the inter-regional covariation of the gray matter volume by Bassett et al. using MRI data from 259 healthy volunteers [21]. In this data classical divisions of the cortex (multimodal, unimodal and transmodal) showed distinct topological distributes. Diffusion imaging is a relatively new MRI technique, which can visualize brain white matter fiber tracts *in vivo* [22]–[28], and has been recently used to investigate human brain anatomical networks. Hagmann et al. made the first attempt by applying diffusion spectrum imaging to two healthy volunteers and was thus the first to confirm small-world topology in the anatomical networks of individual brains [29]. They further extended their investigation into the dense network of cortico-cortical axonal pathways and revealed a structural core in the human cerebral cortex [30]. Another study performed by Iturria-Medina et al. established a weighted anatomical network for individual brains using diffusion tensor imaging (DTI) and graph theory; they also found small-world properties of the networks across 20 subjects [31]. However, their approach will sometimes result in assigning a nonzero connection probability value to brain region pairs which are unlikely to be connected (e.g., left frontal and right occipital cortex) [31]. In a recent study by Gong et al. [32], a macro scale anatomical network was established across 80 healthy volunteers using diffusion tensor tractography (DTT). The entire cerebral cortex was subdivided into 78 regions, not including the subcortical structures, using automated anatomical labeling (AAL). Their findings suggested prominent small-world attributes which are generally compatible with the findings of previous studies. However, only one group-based binary network was generated from all subjects using their approach, leaving the investigation of individual brains and the construction of weighted brain networks unstudied.

In the present study, we tested the hypothesis that individual intelligence is associated with the individual's brain structural organization. Specifically, higher intelligence test scores correspond to a higher global efficiency of the individual's brain anatomical network. We performed our study on 79 healthy young adults, basically using the DTT method proposed by Gong et al. [32] with some modifications to allow the method to fit our goal. First, we constructed a binary anatomical network of the individual brain of each subject using a modified method, in which subcortical structures (i.e. the thalamus) were included and a robust algorithm for fiber tracking was employed. Secondly, we developed the binary networks into weighted ones by introducing an appropriate index to achieve a more complete picture for our investigation. Thirdly, topological properties of the binary and the weighted anatomical networks of each subject were calculated and used for the small-world evaluation. Fourthly, depending on their IQ tests scores, all healthy adults were divided into general intelligence (GI) and high intelligence (HI) groups, and a two-sample *t-test* of network properties was performed between the two groups. Finally, partial correlation analyses were performed between the IQ scores and the topological properties of brain anatomical networks across all subjects while controlling for age and gender. To obtain convergent evidence from the test of our hypothesis, both inter-group comparisons and partial correlation analyses were performed on the binary and the weighted networks; we also reproduced our investigation utilizing different brain parcellation schemes for network construction as well as different indices for weighted network construction.

## Results

### Topological properties of the brain anatomical network

**Figure 1:**
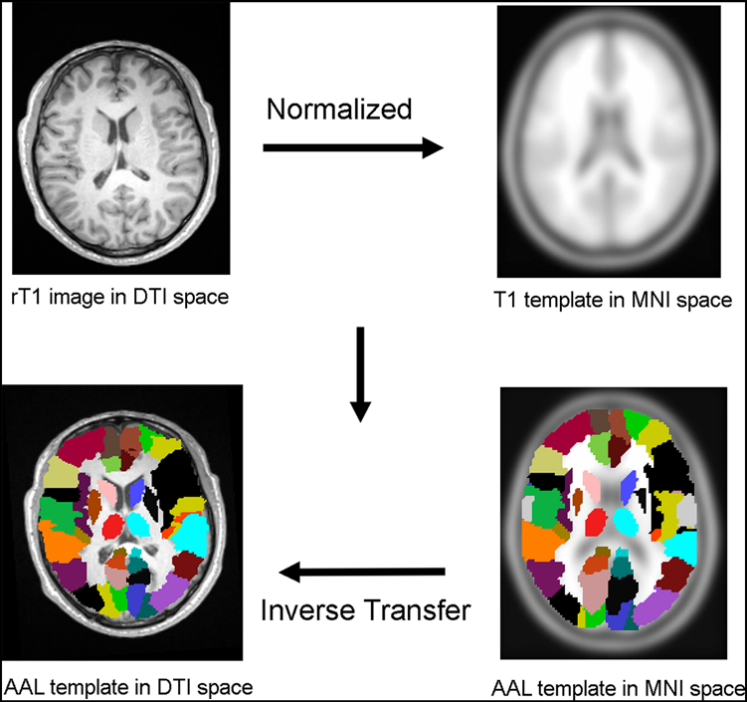
Schematic representation of the transformation of the AAL template into individual DTI space. Both the T1 template and the AAL template showed in the right column are in the MNI space, with image dimensions of 181 mm×217 mm×181 mm and voxel dimensions of 1 mm×1 mm×1 mm. Both the rT1 image and the transformed AAL template overlaid on it showed in the left column are in the DTI native space of one randomly selected individual, with image dimensions of 256 mm×256 mm×45 mm and voxel dimensions of 1 mm×1 mm×3 mm. The homologous brain regions in AAL template were coded in different colors because the areas in the left and right hemispheres were considered separately.

**Figure 2:**
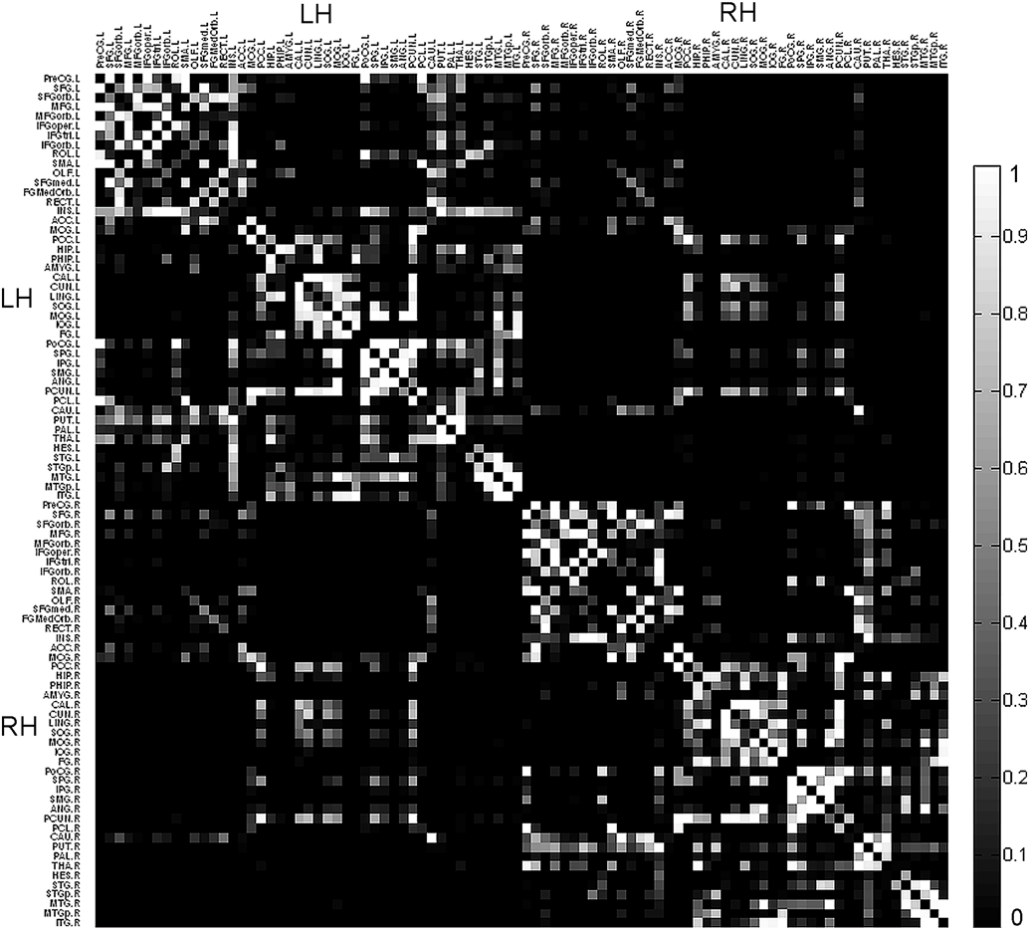
Mean map of the binary connectivity matrixes averaged across the 79 subjects. A 90×90 symmetric matrix in which the *x* and *y* axes correspond to the regions listed in Table 1 (labeled by the abbreviations defined in Table 1) and each entry represents the percentage of subjects that have a connection between the corresponding pair of brain regions. The value of each entry ranged from 0 (black color on the map), indicating that no subject showed a connection between the corresponding pair of brain regions, to 1 (white color in the map), indicating that the two regions were connected in all subjects. The regions in the left and right hemispheres are ordered separately. Abbreviations: LH, Left Hemisphere; RH, Right Hemisphere.

**Figure 3:**
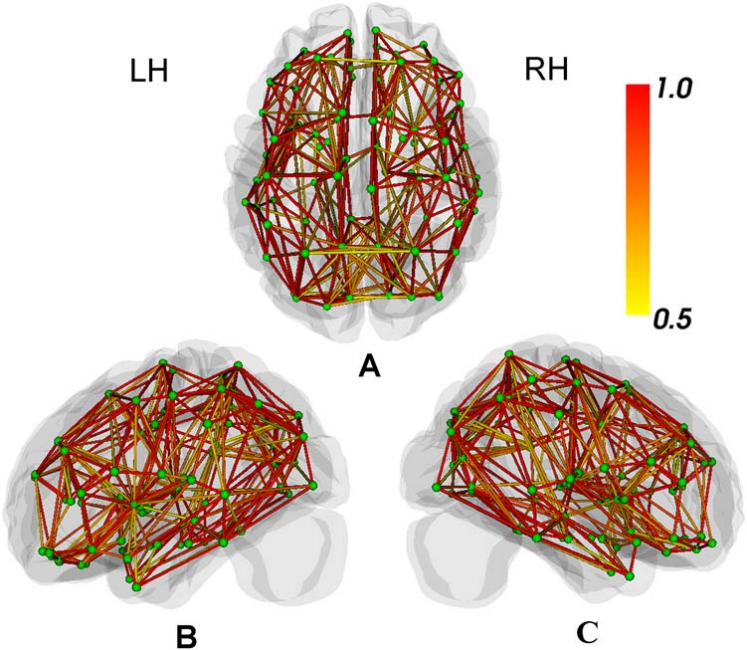
3D presentation of the binary connectivity matrixes averaged across the 79 subjects. (A), (B) and (C): A 3D presentation of the network in anatomical space, in which the green points correspond to the 90 AAL regions defined in Table 1 and the lines correspond to the connection between corresponding pairs of brain regions. The colors of the lines represents the percentage of subjects that have a connection between the corresponding pair of brain regions, ranging from 0.5 (yellow color), indicating that at least half of the subjects showed a connection between the corresponding pair of brain regions, to 1 (red color), indicating that the two regions were connected in all subjects. Abbreviations: LH, Left Hemisphere; RH, Right Hemisphere.

**Figure 4:**
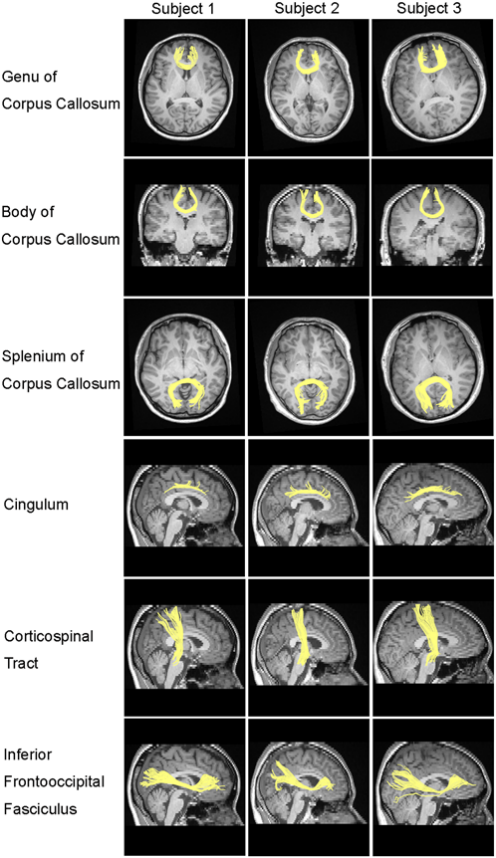
Six well-known major white matter tracts reconstructed in three randomly selected subjects. Please note that the fiber bundles showed here may be only parts of a specific major white matter tract, rather than the entire tract.

We successfully constructed binary and weighted anatomical networks for each of the 79 subjects in the form of symmetric connectivity matrixes using our method (see Materials and Methods, Fig. 1, Tables 1 and 2). Figures 2 and 3 show the mean map which was obtained by averaging across the binary connectivity matrixes of all 79 subjects (Fig. 2) as well as a 3D representation of the network in anatomical space (Fig. 3 A, B and C). The network is primarily comprised of intra-hemispheric connections with a few major inter-hemispheric connections. This connection pattern is generally comparable with previous brain anatomical network studies utilizing MRI and diffusion imaging data [20], [30]–[32]. Please note that we constructed the network showed in Figs. 2 and 3 using a threshold value of 3 (see Materials and Methods). In addition, six well-known white matter fiber tracts - the genu of the corpus callosum (CC), the body of the CC, the splenium of the CC, the cingulum, the corticospinal tract and the inferior frontooccipital fasciculus - were further constructed in three randomly selected subjects utilizing our fiber tracking method and are presented in Fig. 4. We used the AAL regions as seed regions and some extra ROIs as filters which are necessary for correctly reconstructing the six fiber tracts. In detail, the filter ROIs for the corpus callosum were placed on the midsagittal planes; the ROIs for the cingulum were placed through the genu-trunk junction and the trunk-splenium junction of the corpus callosum in coronal planes; the ROIs for the corticospinal tract were placed in the posterior limb of the internal capsule and the pre- and postcentral gyri respectively; and the ROIs for inferior frontooccipital fasciculus included large part of the entire frontal and occipital lobes [33],[34]. The trajectories of these major white matter tracts are consistent with the existing anatomical knowledge-base [35] as well as with a previous DTI study [36]. This consistency with anatomical and DTI information may provide further support for the validation of our constructed network.

**Table 1:** Cortical and sub-cortical regions defined in the AAL template.

Region name	Abbreviation	Region name	Abbreviation
Precentral	PreCG	Lingual	LING
Frontal_Sup	SFG	Occipital_Sup	SOG
Frontal_Sup_Orb	SFGorb	Occipital_Mid	MOG
Frontal_Mid	MFG	Occipital_Inf	IOG
Frontal_Mid_Orb	MFGorb	Fusiform	FG
Frontal_Inf_Oper	IFGoper	Postcentral	PoCG
Frontal_Inf_Tri	IFGtri	Parietal_Sup	SPG
Frontal_Inf_Orb	IFGorb	Parietal_Inf	IPG
Rolandic_Oper	ROL	SupraMarginal	SMG
Supp_Motor_Area	SMA	Angular	ANG
Olfactory	OLF	Precuneus	PCUN
Frontal_Sup_Medial	SFGmed	Paracentral_Lobule	PCL
Frontal_Mid_Orb	FGMedOrb	Caudate	CAU
Rectus	RECT	Putamen	PUT
Insula	INS	Pallidum	PAL
Cingulum_Ant	ACC	Thalamus	THA
Cingulum_Mid	MCG	Heschl	HES
Cingulum_Post	PCC	Temporal_Sup	STG
Hippocampus	HIP	Temporal_Pole_Sup	STGp
ParaHippocampal	PHIP	Temporal_Mid	MTG
Amygdala	AMYG	Temporal_Pole_Mid	MTGp
Calcarine	CAL	Temporal_Inf	ITG
Cuneus	CUN		

Abbreviations: AAL, Automated Anatomical Labeling.

**Table 2:** Topological properties of binary anatomical networks constructed on the individual brains of all 79 subjects using five different threshold values.

Threshold value	SOBCC group mean (SD)	E group mean (SD)	Cp group mean (SD)	Lp group mean (SD)	γ group mean (SD)	λ group mean (SD)	E_glob group mean (SD)
1	90 (0.16)	1185 (±101)	0.52 (±0.01)	2.32 (±0.09)	1.83 (±0.15)	1.11 (±0.02)	0.50 (±0.02)
2	90 (0.35)	921 (±85)	0.50 (±0.02)	2.60 (±0.11)	1.82 (±0.12)	1.13 (±0.02)	0.45 (±0.02)
**3**	**90 (0.47)**	**785 (±79)**	**0.49 (±0.02)**	**2.81 (±0.14)**	**2.07 (±0.20)**	**1.14 (±0.03)**	**0.42 (±0.02)**
4	89 (0.66)	694 (±74)	0.48 (±0.02)	2.99 (±0.17)	2.07 (±0.21)	1.17 (±0.03)	0.40 (±0.02)
5	89 (0.75)	625 (±68)	0.47 (±0.02)	3.15 (±0.19)	2.13 (±0.23)	1.18 (±0.04)	0.39 (±0.02)

Abbreviations: SOBCC, Size of Biggest Connected Component; SD, Standard deviation.

Notes: E, Cp, Lp denote the number of edges, average clustering coefficient and mean shortest path length of the network respectively. γ and λ denote the small-world properties of the network. E_glob denotes the absolute global efficiency of the network. Detailed definitions can be found in the Materials and Methods section. The values are showed in the form of group means (±SD), which were obtained by averaging across all 79 subjects.

Network measures included the total number of edges 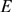, absolute clustering coefficient 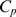, mean characteristic path length 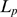 and global efficiency 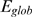 of the network as well as the small-world indices 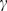 and 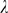 (see Materials and Methods). The average value of these topological properties of the binary and the weighted networks across all the 79 subjects are listed in Table 3 along with the results of previous studies on functional and anatomical human brain networks at a macro scale level [15],[16],[20],[31],[32]. Our results are very compatible with these previous findings. In particular, a prominent small-world attribute was consistently observed in the binary networks of all 79 healthy volunteers. In addition, we examined the hub regions and degree distributions of the binary networks we constructed. These examinations showed consistent results with previous studies of functional or anatomical networks, providing further support for our current study (Details can be found in Text S3).

**Table 3:** Topological properties including small-world indices of human brain networks in the current study and previous studies.

Brain networks	N	Cp	Lp	γ	λ	E_glob
**Anatomical network (Current study, binary)**	**90**	**0.49 (±0.02)**	**2.81 (±0.14)**	**2.07 (±0.20)**	**1.14 (±0.03)**	**0.42 (±0.02)**
**Anatomical network (Current study, weighted)**	**90**	**0.55 (±0.02)**	**0.15 (±0.03)**	**2.20 (±0.21)**	**1.27 (±0.08)**	**11.04 (±2.10)**
Anatomical network (Gong *et al.* 2008)	78	0.49	2.32	4.07	1.15	Not reported
Anatomical network (Iturria-Medina *et al.* 2008)	90	Not reported	Not reported	1.85	1.12	Not reported
Morphological network (He *et al.* 2007)	54	0.30	3.05	2.36	1.15	Not reported
Functional network (Achard *et al.* 2006)	90	0.53	2.49	2.37	1.09	Not reported
Functional network (Salvador *et al.* 2005)	45	0.25	2.82	2.08	1.09	Not reported

Notes: N, Cp, Lp denote the number of nodes, average clustering coefficient and mean shortest path length of the network respectively. γ and λ denote the small-world properties of the network. E_glob denotes the absolute global efficiency of the network. Detailed definitions can be found in the Materials and Methods section. The values from our current study are showed in the form of group means (±SD), which were obtained by averaging across all 79 subjects.

### Different network properties between GI and HI groups

As shown in Table 4, significant differences in network properties were found between the GI and HI groups by a two-sample *t-test* (see Materials and Methods): 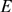 was significantly larger in the HI group; the 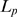 of the binary and the weighted networks was significantly shorter in HI group; the 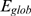 of the binary and the weighted networks was significantly higher in HI group; no significant difference in 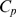 was observed between the two groups in the binary and weighted networks. In most cases, the weighted networks showed a much smaller *P*-value than the binary networks, suggesting that the differences in network properties between these two groups were more significant in the weighted networks. Please note that these results were observed using a threshold value of 3 to construct the network (see Materials and Methods). To explore the dependence of our results on our choice of threshold, we reproduced the two-sample *t-test* between the GI and HI groups on binary and weighted networks that we constructed using five different threshold values ranging from 1 to 5. Similar results were consistently observed, suggesting that our findings are relatively robust (Table 4).

**Table 4:** Two-sample *t-test* on properties of binary and weighted networks between GI and HI groups.

Threshold value	Topological properties		Value, group mean (SD)		*P*- value (Two-sample *t-test*) (Equal variances assumed)
			GI (n = 42)	HI (n = 37)	GI *vs* HI
1	E		1160.95 (95.51)	1211.51 (101.19)	0.025
	Cp	Binary	0.52 (0.01)	0.52 (0.01)	0 .213
		Weighted	0.60 (0.02)	0.61 (0.01)	0.063
	Lp	Binary	2.34(0.09)	2.30(0.07)	0.019
		Weighted	0.16 (0.03)	0.14 (0.02)	<0.001 *
	E_glob	Binary	0.50(0.02)	0.51(0.02)	0.019
		Weighted	10.28 (1.74)	11.79 (2.20)	0.001 *
2	E		899.05 (78.61)	946.11 (85.65)	0.013
	Cp	Binary	0.50 (0.02)	0.50 (0.02)	0 .217
		Weighted	0.57 (0.02)	0.57 (0.02)	0.162
	Lp	Binary	2.63 (0.11)	2.57 (0.10)	0.015
		Weighted	0.16 (0.03)	0.14 (0.02)	<0.001 *
	E_glob	Binary	0.45 (0.02)	0.46 (0.02)	0.012
		Weighted	10.31 (1.74)	11.83 (2.23)	0.001 *
**3**	**E**		**760.86 (70.52)**	**812.16 (79.85)**	0.003 *
	**Cp**	**Binary**	**0.48 (0.01)**	**0.49 (0.02)**	**0.030**
		**Weighted**	**0.54 (0.02)**	**0.55 (0.02)**	**0.014**
	**Lp**	**Binary**	**2.85 (0.14)**	**2.76 (0.13)**	0.004 *
		**Weighted**	**0.16 (0.02)**	**0.14 (0.02)**	<0.001 *
	**E_glob**	**Binary**	**0.42 (0.02)**	**0.43 (0.02)**	0.003 *
		**Weighted**	**10.33 (1.74)**	**11.84 (2.21)**	0 .001 *
4	E		670.14 (66.01)	721.08 (74.22)	0.002 *
	Cp	Binary	0.47 (0.02)	0.49 (0.02)	0.018
		Weighted	0.52 (0.02)	0.54 (0.02)	0.006 *
	Lp	Binary	3.04 (0.17)	2.94 (0.15)	0.006 *
		Weighted	0.15 (0.02)	0.13 (0.02)	0.001 *
	E_glob	Binary	0.40 (0.02)	0.41 (0.02)	0.004 *
		Weighted	10.40 (1.73)	11.91 (2.27)	0.001 *
5	E		601.86 (60.05)	650.86 (68.36)	0.001 *
	Cp	Binary	0.47 (0.02)	0.47 (0.02)	0.183
		Weighted	0.51 (0.02)	0.52 (0.02)	0.034
	Lp	Binary	3.21 (0.19)	3.01 (0.17)	0.004 *
		Weighted	0.15 (0.02)	0.13 (0.02)	<0.001 *
	E_glob	Binary	0.38 (0.02)	0.39 (0.02)	0.003 *
		Weighted	10.44 (1.72)	11.97 (2.30)	0.001 *

Significance was set at 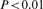 with equal variances assumed. The results for a threshold of 3 are bolded, as these were chosen for inclusion in the paper.

Abbreviations: GI, General Intelligence; HI, High Intelligence.

### Relationship between intelligence test scores and network properties

**Figure 5:**
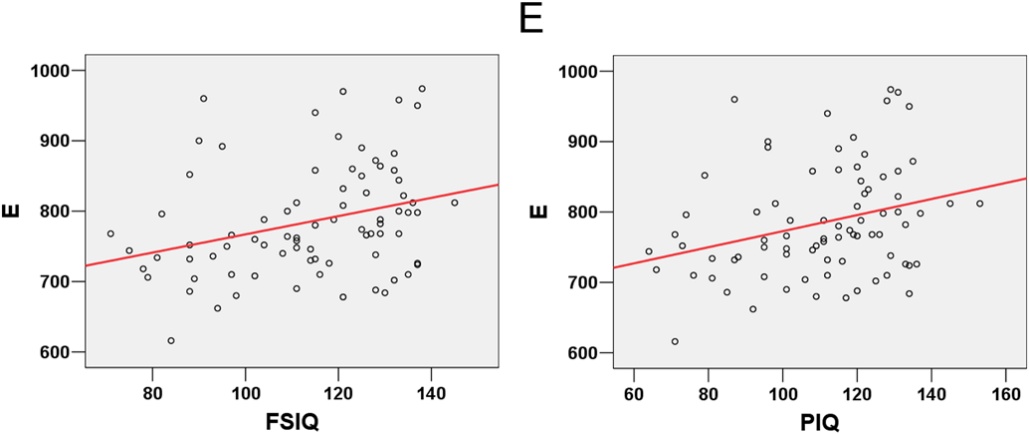
Significant partial correlation between the total number of edges and intelligence tests scores. 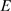
 was found to be positively correlated to FSIQ and PIQ.

**Figure 6:**
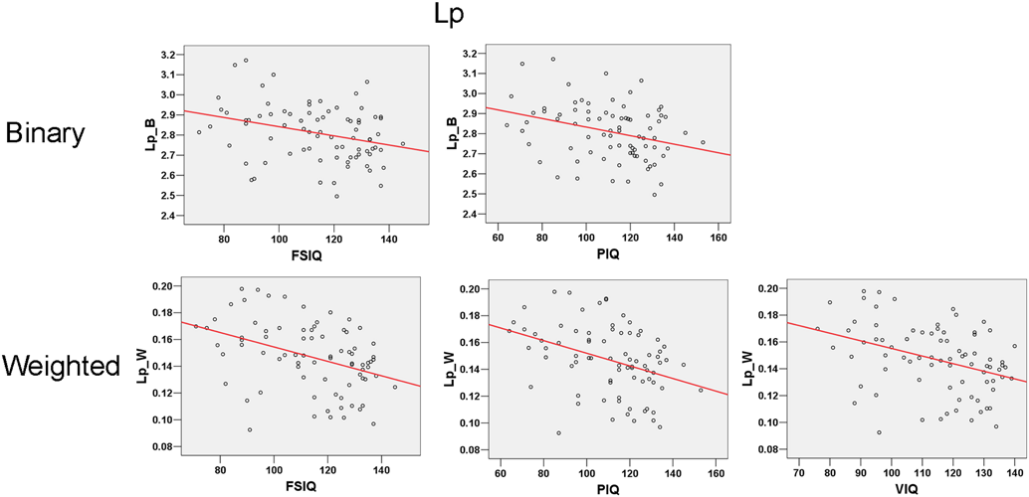
Significant partial correlation between Lp and intelligence tests scores. In the case of binary networks, 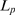 was found to be negatively correlated to FSIQ and PIQ; and in the case of weighted networks, 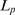 was found to be negatively correlated to FSIQ, PIQ and VIQ.

**Figure 7:**
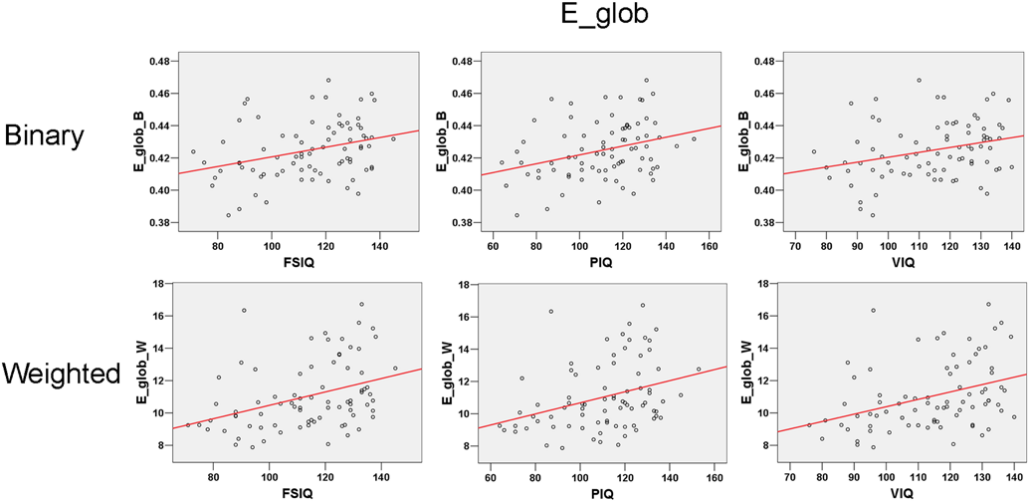
Significant partial correlation between E_glob and intelligence tests scores. 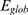
 was found to be positively correlated to FSIQ, PIQ and VIQ in both the binary and weighted networks.

Intelligence test scores included full scale IQ (FSIQ), performance IQ (PIQ) and verbal IQ (VIQ) (see Materials and Methods). As shown in Table 5, significant correlations between the intelligence test scores and the topological properties of the binary and the weighted anatomical brain networks were found by partial correlation analyses in all 79 subjects, when the data were controlled for age and gender (see Materials and Methods): 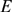 was found to be positively correlated to FSIQ and PIQ (Fig. 5); for the binary networks, 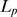 was found to be negatively correlated to FSIQ and PIQ, and for the weighted networks, 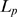 was found to be negatively correlated to FSIQ, PIQ and VIQ (Fig. 6); 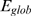 was found to be positively correlated to FSIQ, PIQ and VIQ in the binary and the weighted networks for all subjects (Fig. 7); no significant correlation was found between 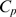 and the intelligence tests scores. In most cases, the weighted networks showed a much larger absolute value of the partial correlation coefficient and a much smaller *P*-value than the binary networks, suggesting that the correlations were stronger and more significant in the weighted networks. Having established that changing the threshold values did not change our overall conclusions, we will use a threshold value of 3 throughout the rest of the Results section.

**Table 5:** Partial correlation between topological properties and intelligence test scores across all subjects while controlling for age and gender.

Threshold value	Topological properties		FSIQ		PIQ		VIQ	
			PCC	*P*-value	PCC	*P*-value	PCC	*P*-value
1	E		0.173	0.132	0.159	0.167	0.167	0.146
	Cp	Binary	0.040	0.730	0.006	0.960	0.068	0.559
		Weighted	0.114	0.322	0.071	0.537	0.146	0.206
	Lp	Binary	−0.192	0.094	−0.179	0.120	−0.184	0.108
		Weighted	−0.312	0.006 *	−0.289	0.011 *	−0.297	0.009 *
	E_glob	Binary	0.189	0.099	0.176	0.125	0.182	0.114
		Weighted	0.302	0.008 *	0.273	0.016 *	0.291	0.010 *
2	E		0.187	0.104	0.186	0.106	0.166	0.149
	Cp	Binary	0.123	0.286	0.110	0.341	0.121	0.294
		Weighted	0.131	0.256	0.111	0.336	0.134	0.245
	Lp	Binary	−0.198	0.085	−0.206	0.073	−0.172	0.135
		Weighted	−0.338	0.003 *	−0.325	0.004 *	−0.314	0.005 *
	E_glob	Binary	0.200	0.081	0.206	0.073	0.176	0.125
		Weighted	0.304	0.007 *	0.277	0.015 *	0.293	0.010 *
**3**	**E**		**0.242**	0.034 *	**0.242**	0.034 *	**0.213**	**0.063**
	**Cp**	**Binary**	**0.138**	**0.231**	**0.117**	**0.311**	**0.142**	**0.218**
		**Weighted**	**0.204**	**0.075**	**0.183**	**0.111**	**0.200**	**0.081**
	**Lp**	**Binary**	**−0.262**	0.021 *	**−0.275**	0.016 *	**−0.221**	**0.053**
		**Weighted**	**−0.359**	0.001 *	**−0.342**	0.002 *	**−0.332**	0.003 *
	**E_glob**	**Binary**	**0.264**	0.021 *	**0.272**	0.017 *	**0.227**	0.047 *
		**Weighted**	**0.308**	0.006 *	**0.281**	0.013 *	**0.296**	0.009 *
4	E		0.254	0.026 *	0.264	0.020 *	0.217	0.058
	Cp	Binary	0.175	0.127	0.133	0.250	0.197	0.085
		Weighted	0.218	0.057	0.179	0.119	0.233	0.042 *
	Lp	Binary	−0.270	0.017 *	−0.285	0.012 *	−0.230	0.044 *
		Weighted	−0.357	0.001 *	−0.330	0.003 *	−0.338	0.003 *
	E_glob	Binary	0.272	0.017 *	0.284	0.012 *	0.233	0.041 *
		Weighted	0.306	0.007 *	0.275	0.015 *	0.297	0.009 *
5	E		0.265	0.020 *	0.255	0.025 *	0.243	0.033 *
	Cp	Binary	0.017	0.884	−0.015	0.898	0.040	0.727
		Weighted	0.111	0.336	0.076	0.513	0.129	0.264
	Lp	Binary	−0.275	0.015 *	−0.287	0.011 *	−0.237	0.038 *
		Weighted	−0.365	0.001 *	−0.339	0.003 *	−0.347	0.002 *
	E_glob	Binary	0.277	0.015 *	0.279	0.014 *	0.246	0.031 *
		Weighted	0.309	0.006 *	0.277	0.015 *	0.300	0.008 *

Significance was set at *P*<*0.05*. The results for a threshold of 3 are bolded, as these were chosen for inclusion in the paper.

Abbreviations: FSIQ, Full Scale IQ; PIQ, Performance IQ; VIQ, Verbal IQ; PCC, Partial Correlation Coefficient.

To further localize the association with intellectual performance, the local efficiency (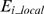) of each node region was calculated for each subject (see Materials and Methods). As shown in Tables 6 and 7, when we controlled for age and gender, we found significant correlations (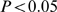, uncorrected) using partial correlation analyses performed across all subjects between their intelligence test scores and the local efficiency (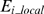) of multiple brain regions, which were located in widely-distributed areas across the brain. These involved cortical areas in the parietal, temporal, occipital and frontal lobes as well as subcortical structures such as the thalamus, amygdala and hippocampus.

**Table 6:** Brain regions that showed significant correlations between the local efficiency and intelligence test scores in binary networks across all subjects while controlling for age and gender.

Brain regions (Abbreviation)	FSIQ		PIQ		VIQ	
	PCC	*P*-value	PCC	*P*-value	PCC	*P*-value
PoCG _L	0.289	0.011 *	0.242	0.034 *	0.303	0.007 *
STGp _R	−0.265	0.020 *	−0.255	0.025 *	−0.248	0.030 *
MCG _R	0.256	0.025 *	0.190	0.098	0.286	0.012 *
AMYG _R	0.249	0.029 *	0.262	0.021 *	0.232	0.042 *
MOG _L	0.215	0.061	0.282	0.013 *	0.130	0.260
MTGp _R	0.211	0.066	0.290	0.010 *	0.106	0.357
MFG _R	0.180	0.116	0.249	0.029 *	0.091	0.433

The threshold value was set at *P*<*0.05* for significance (uncorrected). The abbreviations of brain regions were defined in Table 1. “L” indicates that the region was located in left hemisphere; “R” stands for right hemisphere.

Abbreviations: FSIQ, Full Scale IQ; PIQ, Performance IQ; VIQ, Verbal IQ; PCC, Partial Correlation Coefficient.

**Table 7:** Brain regions that showed significant correlations between the local efficiency and intelligence test scores in weighted networks across all subjects while controlling for age and gender.

Brain regions (Abbreviation)	FSIQ		PIQ		VIQ	
	PCC	*P*-value	PCC	*P*-value	PCC	*P*-value
PoCG _L	0.349	0.002 *	0.345	0.002 *	0.313	0.006 *
IFGoper _R	0.333	0.003 *	0.274	0.016 *	0.343	0.002 *
CUN _L	0.303	0.007 *	0.253	0.026 *	0.310	0.006 *
PCUN _R	0.283	0.013 *	0.266	0.019 *	0.262	0.022 *
PCC _R	0.275	0.016 *	0.245	0.032 *	0.270	0.017 *
IFGorb _L	0.271	0.017 *	0.194	0.092	0.324	0.004 *
MOG _L	0.258	0.023 *	0.320	0.005 *	0.168	0.143
SOG _R	0.257	0.024 *	0.272	0.017 *	0.216	0.059
MOG _R	0.255	0.025 *	0.267	0.019 *	0.208	0.070
PreCG _L	0.252	0.027 *	0.223	0.051	0.262	0.021 *
MTG _L	0.247	0.030 *	0.212	0.064	0.251	0.028 *
THA _R	0.244	0.033 *	0.168	0.145	0.278	0.014
PAL _R	0.240	0.036 *	0.216	0.059	0.251	0.028 *
SFGorb _L	0.240	0.036 *	0.158	0.171	0.288	0.011 *
MFGorb _L	0.237	0.038 *	0.174	0.129	0.266	0.019 *
PAL _L	0.234	0.040 *	0.166	0.148	0.269	0.018 *
CUN _R	0.222	0.052	0.225	0.049 *	0.179	0.119
RECT _L	0.220	0.054	0.143	0.213	0.268	0.018 *
HIP _R	0.208	0.069	0.267	0.019 *	0.137	0.236

The threshold value was set at *P*<*0.05* for significance (uncorrected). The abbreviations of brain regions were defined in Table 1. “L” indicates that the region was located in the left hemisphere; “R” stands for the right hemisphere.

Abbreviations: FSIQ, Full Scale IQ; PIQ, Performance IQ; VIQ, Verbal IQ; PCC, Partial Correlation Coefficient.

### Convergent evidence from comprehensive analyses

We reproduced our investigations utilizing different brain parcellation schemes for network construction (see Text S1) as well as different indices for weighted network construction (see Text S2). In each of these situations, we calculated the topological properties of brain networks for small-world evaluation and performed statistical analyses, including inter-groups comparisons and partial correlation analyses between IQ scores and brain network properties across all subjects as well. The results of these analyses showed that, in most of the tested situations, prominent small-world attributes were consistently observed across all the 79 subjects (see Text S1). More importantly, significantly higher global efficiencies of the brain networks were consistently observed in the HI group (see Text S1 and Text S2), and significant correlations were consistently found between specific IQ scores and brain network properties (see Text S1 Text S2 as well as Figs.S1, S2, S3, S4 and S5). In particular, higher intellectual performance corresponds to better global efficiency of the brain networks. These comprehensive analyses provide convergent evidence for the validity of our findings.

## Discussion

In this study, we successfully constructed binary and weighted anatomical networks for individual brains from 79 healthy young adults using a DTT method. Network topological properties were analyzed and prominent small-world attributes were found. These findings are in accordance with the findings of previous human brain network studies that were done at a macro scale level [15],[16],[20],[31],[32]. More importantly, we found convergent evidence supporting our hypothesis that individual differences in intelligence are associated with the structural organization of the brain. Significant differences in network properties were observed between the GI and HI groups. Specifically, significant correlations were found between intelligence tests scores and global network topological properties from all subjects while controlling for age and gender. To the best of our knowledge, this is the first study that investigated the relationship between intelligence and the brain anatomical network utilizing the DTT method and supported the concept that complex brain network topology parameters have cognitive significance.

### Topological properties of the brain anatomical network

#### Efficient small-world brain anatomical network

After its introduction by Watts and Strogatz [18], the small-world attribute has been found in numerous complex networks, including social, economic and biological networks. It is characterized by a high local clustering of connections between neighboring nodes and short path lengths between any pair of nodes [37]. The cortical networks of other mammalian brains [12],[38] as well as functional and structural human brain networks exhibit small-world properties [13]–[16], [20], [29]–[32],[39]. In keeping with these earlier findings, a salient small-world attribute was observed consistently in the individual brains of all our healthy volunteers using our network construction method based on DTT. However, it should be noted that the mean value of the small-world index, 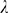, for the weighted networks using the number of existing fibers as the weighted index was considerably higher than the mean value of 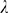 for the binary networks and for the weighted networks obtained using an average fractional anisotropy (FA) as the weighted index (see Text S1). Thus our network construction method seems to indicate that the number of existing fiber bundles may not be ideally suited for describing all aspects of the topological properties of the brain network. This could result from a variety of factors such as the limited resolution of DTI, or the inadequate capacity of the deterministic fiber tracking method we employed in dealing with the “fiber crossing” problem, or the procedure used for generating the random network when calculating the small-world index. The question of how best to weight the connectivity between two brain regions remains open. Nevertheless, although our current analysis of brain anatomical networks may be not completely comparable to previous investigations due to differences in species and network construction approaches, our results give good support to the common finding that small-world topology is a fundamental principle of the structural and functional organization of complex brain networks [32],[40].

#### Hubs and degree distribution

We identified hub regions, which have been identified by previous studies of functional or anatomical networks [16],[20],[30],[32],[39], such as the PCUN (see Text S3). We also found that the distribution of the node degrees followed an exponentially truncated power-law (see Text S3 and Fig.S6). These findings appear to provide further support for the validation of our current study (extended details can be found in Text S3).

In general, the topological properties of the brain anatomical network constructed in our current study are compatible with the findings of previous human brain network studies. However, some discrepancies exist between our results and previous findings, such as the exact values of the topological properties including the small-world indices. These discrepancies may be due to differences in data types and analytical methods.

### Relationship between intelligence and brain structural organization

In this study, global efficiency of the brain anatomical network was higher in the HI groups than in the GI groups, and positive correlations between intelligence tests scores and the global efficiency of the networks were found in all the healthy young adults while controlling for age and gender. These findings were consistently observed in the different situations we tested, including the binary and the weighted networks we constructed, the different brain parcellation schemes we employed (see Text S1) and the various indices we used for weighted network construction (see Text S2).

Many previous studies have related intelligence to different structural and functional properties of the brain. Positive correlations between IQ and total brain volume have been reported by several research teams who used structural imaging techniques on different populations with different scan protocols and different intelligence measures [41]–[46]. Utilizing voxel-based morphometry methods, recent studies have revealed correlations between IQ and certain specific brain regions involving the frontal, parietal, temporal and occipital lobes [3]–[5], [47]–[50]. Several previous functional imaging studies, using intellectually demanding tasks ranging from working memory to a variety of verbal and non-verbal reasoning, have also shown that people who performed well on intelligence related tests recruited multiple brain regions [1],[2],[9],[51]. Although none of these previous studies investigated the issue from the perspective of brain networks, they can nonetheless provide support for our current findings. Partial correlation analyses performed across all subjects while controlling for age and gender revealed significant correlations between intelligence test scores and the local efficiency (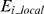) of multiple brain regions, including cortical areas located in the parietal, temporal, occipital and frontal lobes as well as subcortical structures such as the thalamus, amygdale and hippocampus (Tables 6 and 7). Please note that the significance level for our partial correlation analyses of the local efficiency (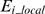) was set at 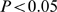 and was uncorrected for multiple comparisons across all the 90 brain regions. An even higher level of significance might be able to be achieved in future studies by including more subjects. However, although the interpretation of our results must be cautious, our findings appear to provide new evidence for the biological basis of intelligence from a network perspective. In particular, in one recent review of 37 neuroimaging studies associated with the neural basis of intelligence [11], Jung and Haier found that individual differences in intelligence were closely related to variations in a distributed brain network which included multiple brain regions located in the dorsolateral prefrontal cortex, the inferior and superior parietal lobe, the anterior cingulate, the temporal and the occipital lobes. Our investigations may provide evidence for their findings from a brain anatomical network perspective, and more importantly, our findings may indicate that the efficient organization of the brain anatomical network may be important for individual intellectual performance.

In a recent study performed by our group [34], a partial correlation analysis on the same 79 healthy volunteers together with 15 mental retardation patients controlling for age and gender showed that FSIQ scores were significantly correlated with the FA value of the bilateral uncinate fasciculus, the genu and truncus of the corpus callosum, the bilateral optic radiation and the left corticospinal tract. Significant correlation was also found between the FSIQ scores and the FA of the right UF when further controlling for group identity between patient and normal control [34]. The findings of this earlier research provide structural evidence for our current investigation by showing that the integrity of the major white matter bundles, which was measured by the FA value, may be an important biological basis for human intelligence. The results of our current study show that higher intelligence test scores are related to a larger global efficiency (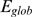) of the brain anatomical network (Table 5 and Fig. 7), which may indicate better parallel information transfer in the brain [52]. According to the DTT method, in which the propagation of fiber tracking depends on white matter integrity as measured by the FA value, we may speculate that the more efficient network organization associated with better intellectual performance may relate to increased white matter integrity, not only in the major fiber bundles investigated in our previous study but also in the white matter connectivity across the whole brain. Our findings support the previous finding that cognitive processes are dependent upon the fidelity of the underlying white matter to facilitate the rapid and error-free transmission of data between different brain regions [11]. In another resting state functional MRI study on a subset of the same 79 healthy adults (59 subjects) performed by our group [6], brain regions in which the strength of functional connectivity significantly correlated with intelligence scores were distributed in the frontal, parietal, occipital and limbic lobes. This gives increased credence to our current study by supporting a network view of intelligence from functional imaging evidences, thus revealing that brain activity may be relevant to differences in intelligence even in the resting state [6].

Subjects with higher IQ scores consistently showed more edges (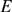) and shorter characteristic path lengths (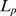) in the various situations which we tested. This is consistent with previous findings that short paths in brain networks assure effective integrity or rapid transfer of information between and across remote regions that are believed to constitute the basis of cognitive processes [12]. A previous study performed by Kaiser and Hilgetag [53] demonstrated that neural systems are not optimized exclusively for minimal global wiring length, but for a variety of factors including the minimization of processing steps. Although not completely comparable in data types and analysis methods, our finding of shorter characteristic path lengths (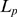) in the subjects with higher IQ scores may reflect fewer signal processing steps between brain regions.

As reviewed by Roth and Dicke [54], no universally accepted definition of animal intelligence exists; nor has any procedure for measuring it come to dominate the field. One view that has emerged from previous studies of comparative and evolutionary psychologists and cognitive ecologists is that animal intelligence can be defined as the degree of mental or behavioral flexibility resulting in novel solutions, either in the wild or in the laboratory [54]–[57]. According to review studies of previous intelligence investigations [11],[54], various brain properties such as brain volume, relative brain volume and encephalization quotient have been assumed to be relevant for intelligence. However, although humans are generally considered to be the most intelligent species, they do not have the largest brain or cortex, either in absolute or relative terms. But they do have the largest number of cortical neurons and a relatively high conduction velocity between those neurons, which appears to correlate better with intelligence as the basis for information processing capacity [54]. Significantly, myelinated cortical fibers are relatively thin in elephants and cetaceans, but particularly thick in primates [58],[59], contributing to a better conduction velocity. This supports the idea that an increase in information processing capacity is of great importance for intelligence [54]. In our study, intelligence test scores were found to be significantly correlated to the complex brain network topological properties derived from a fiber tracking method based on DTI. Our results appear to support previous findings since DTI is currently the only noninvasive brain imaging technique that can explore the structure of white matter *in vivo* and provide information about the white matter integrity of cortical fibers, a topic which is obviously closely related to fiber myelination [28],[60],[61]. However, more extensive future analyses are necessary to clarify more clearly the relationship between the complex brain network topological parameters that we calculated and the conduction velocity between neurons and to determine how these are related to the information processing capacity of the human brain.

In conclusion, we successfully constructed binary and weighted anatomical networks of the individual brains of 79 healthy adults. These networks showed topological properties that included a prominent small-world attribute that was quite comparable with the findings of previous human brain network studies. More importantly, extensive analysis consistently revealed significant correlations between intelligence test scores and brain anatomical network properties across all subjects, providing convergent evidence for our hypothesis that a more efficient brain structural organization may be an important biological basis for higher intelligence. Our study may provide new clues for understanding the mechanism of intelligence.

## Materials and Methods

### Subjects

It should be noted that the healthy adults included in this current work have been used in previous studies performed by our group for different purposes [6],[34],[62]. However, we will again present the description of these adults in detail here in order to clearly present our current investigation.

Seventy-nine normal subjects (44 males and 35 females, mean age = 23.8 years, range = 17–33 years) were recruited by advertisement. Each subject was examined using the Chinese Revised Wechsler Adult Intelligence Scale (WAIS-RC) [63]. Across all subjects, the mean FSIQ was 113.7 (range = 71–145); the mean test score of PIQ was 110.6 (range = 64–153); and the mean test score of VIQ was 114.4 (range = 76–140). All subjects were right-handed and Han Chinese in origin.

### Ethics Statement

After a full explanation, all subjects gave voluntary written informed consent according to the standards set by the Ethical Committee of Xuanwu Hospital of Capital Medical University.

### MRI data acquisition and preprocessing

Diffusion tensor images of all the subjects were obtained on a 3.0-T Siemens MRI scanner. A single shot echo planar imaging sequence (TR = 6000 ms, TE = 87 ms) was employed. Diffusion sensitizing gradients were applied along 12 non-collinear directions (b = 1000 s/mm^2^), together with a non-diffusion-weighted acquisition (b = 0 s/mm^2^). An integrated parallel acquisition technique was used with an acceleration factor of 2, which can reduce the acquisition time with less image distortion from susceptibility artifacts. From each subject, 45 axial slices were collected. The field of view was 256 mm×256 mm; the acquisition matrix was 128×128 and zero filled into 256×256; the number of excitations was 3; and the slice thickness was 3 mm with no gap, which resulted in a voxel-dimension of 1 mm×1 mm×3 mm. A 3D T1-weighted image for each subject was obtained using a magnetization prepared rapid gradient echo sequence. The imaging parameters were a field of view of 220 mm×220 mm, TE of 2 s, TR of 2.6 ms, flip angle of 9°, and a voxel-dimension of 1 mm×1 mm×1 mm.

Both the DTI data and T1-weighted data were visually inspected by two radiologists for apparent artifacts arising from subject motion and instrument malfunction. Distortions in the diffusion tensor images caused by eddy currents and simple head motions were then corrected by FMRIB's Diffusion Toolbox (FSL 4.0; http://www.fmrib.ox.ac.uk/fsl). After correction, three-dimensional maps of the diffusion tensor and the FA were calculated using the DtiStudio software [64]. T1-weighted images of each subject were co-registered to the subject's non-diffusion-weighted image (b = 0 s/mm^2^) using the SPM2 package (http://www.fil.ion.ucl.ac.uk/spm), resulting in a co-registered T1 image (rT1) in DTI space.

### Construction of the brain anatomical network

#### Definition of network node

First, we employed the AAL template [65] available with the MRIcro software (http://www.sph.sc.edu/comd/rorden/mricro.html) to segment the cerebral cortex of each subject into 90 regions (45 for each hemisphere with the cerebellum excluded), each representing a node of the network. As shown in Fig. 1, the parcellation process for each subject was conducted in the DTI native space according to the method used by Gong et al. [32]. In detail, each individual co-registered T1 image (rT1) was normalized to the T1 template in Montreal Neurological Institute (MNI) space. The resulting inverse transformation was then used to warp the AAL template from MNI space to the DTI native space in which the discrete labeling values were preserved by using a nearest neighbor interpolation method [32]. Both the normalization and the inverse transformation were implemented using the SPM2 package. The definitions of each cortical or sub-cortical region of the AAL template are listed in Table 1.

To provide more support for our current investigation, we also employed the parcellation scheme used by Gong et al. [32], in which the cerebral cortex of each subject was segmented into 78 regions (39 for each hemisphere with the subcortical structures and cerebellum excluded) using the AAL template. The subsequent analyses were also performed under this different parcellation scheme, with the subcortical structures excluded from examination. For details, please see Text S1.

### Construction of the binary network for an individual brain

Subsequently, DTT was performed on every subject. Seed points were selected as voxels with an FA value greater than 0.3 in each node region [66]. The AAL template is not a pure cortical grey matter mask but includes tissues from both cortical grey matter and subcortical white matter [65]. Selecting seed voxels with the criteria of FA>0.3 in every node region helped to ensure that the trajectories we got originated from the white matter tissue underlying the cortical region or adjacent to subcortical structures. A tensorline tracking algorithm, which approximates the direction of fiber propagation by combining the major eigenvector of the tensor, the vector of previous propagation step and the entire tensor itself [67],[68], was implemented using an in-house program developed in the Matlab 7.0 platform. Several previous studies have demonstrated that tensorline tracking methods can achieve robust and reproducible results for fiber bundles reconstruction [68]–[71]. This was helpful when subcortical structures were included for examination in our current study. The tracking procedure was terminated at voxels with an FA value of less than 0.15 or when the angle between adjacent steps was greater than 45° [66].

Two AAL node regions *i* and *j* were considered to be connected if the reconstructed fiber bundles with two end points located in these two regions respectively were present [32]. However, considering the limited resolution of DTI and the capacity of the deterministic tractography method we employed, there is a risk that some false-positive connections will be included. This possibility may increase if only a few fiber bundles are reconstructed between two node regions. In this situation the apparent connections may be the result of noise. To address this issue, a threshold value for the number of presented fibers was utilized to exclude connections between regions that have too few reconstructed fiber bundles to be certain of their validity. On the other hand, some false-negative connections (that is, connections that are real, but are rejected as false) might be excluded when a relatively large threshold value was used. To determine the most appropriate threshold, we tested values from 1 to 5 and calculated the topological properties for the resultant networks of every subject at each tested value. Based on the results showed in Table 2, we chose a value of 3, which was the highest threshold that maintained the average size of the largest connected component at 90 across all subjects, meaning that the 90 brain regions in the network were all connected at this threshold value in the majority of the 79 subjects. A binary symmetric connectivity matrix was obtained for each subject using the above procedures. Please note that to further examine how dependent the results of our study are on the choice of different threshold values, most of the subsequent statistical analyses were also performed on the topological properties of networks constructed using each of the different threshold values ranging from 1 to 5.

#### Construction of a weighted network for an individual brain

We further developed our investigation into weighted anatomical networks by assigning a weighted index to each entry of the binary network constructed in the previous section. In the human brain network study by Hagmann et al. [29], in which a weighted network for an individual brain was constructed based on the results of fiber tracking, the number of connections between two node regions was employed to weight the edge. Although this previous work was performed at the millimeter scale, whereas ours was at a larger scale, and although it used a diffusion spectrum imaging method than ours, it may still provide a guide for the method of weighting networks that we used in our current study. We employed the number of existing fiber bundles between two connecting brain regions as the connectivity weight, 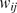, resulting in a weighted symmetric connectivity matrix for each individual.

To investigate other possible weighted indices, we also employed the average FA value of all the reconstructed fiber bundles between two connecting regions and implemented the subsequent inspection of anatomical network properties and statistical analyses on the resulting networks as well. Details can be found in Text S2.

### Anatomical network analyses

#### Graph theoretical analyses of the network topological properties

A complex network can be represented as a graph in which nodes correspond to the elements of the system and arcs to the interactions between them [72]. In our current study, we investigated both a binary anatomical network 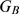 and a weighted one 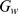, which modeled the anatomical connections between different cortical and subcortical AAL regions for each individual brain. Several topological properties were included for our investigations:

- We used 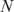 to represent the total number of nodes in the network.
- We used 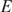 to represent the total number of edges in the network.
- The subgraph 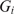 is defined as the set of nodes that are the direct neighbors of the *i*th node. The degree of each node 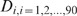 is defined as the number of nodes in 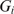. The degree of the network is the average across all the nodes in the graph:
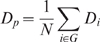

- For the binary network, the absolute clustering coefficient of a node 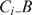 is defined as the ratio of the number of existing connections to the number of all possible connections in the subgraph 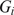:
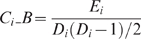
in which 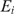 is the number of edges in 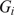 [18],[37]. For a weighted network, the absolute clustering coefficient of a node 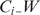 is defined as:
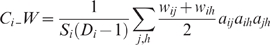
in which 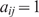 if there is an edge which connects the nodes *i* and *j*; and 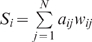 measures the strength of the vertices in terms of the total weight of their connections [73]. The absolute clustering coefficient of the network is the average of all nodes:
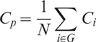
in which 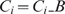 for a binary and 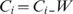 for a weighted network. 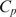 is a measure of the extent of local cliquishness or local efficiency of information transfer of a network [18],[74].
- The mean shortest absolute path length of a node is defined as:
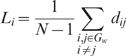
in which 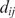 is the shortest absolute path length between the *i*th node and the *j*th node. For a binary network, the length of every edge is 1, and 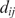 is defined as the number of edges along the shortest path connecting nodes *i* and *j*. For a weighted network, the path with the minimum number of nodes is no longer necessarily the optimal 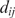 because the length of every edge is associated with the different weight indices between the nodes *i* and *j*. Here we followed the suggestion of Boccaletti et al. [72], and set the length of the edge connecting nodes *i* and *j* inversely proportional to the weight:
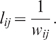
The mean shortest absolute path length of the network is the average across all nodes:
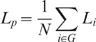
which quantifies the extent of average connectivity or the overall routing efficiency of the network [52].
- The global efficiency of the network 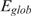 is defined as:
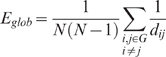
which is the inverse of the harmonic mean of the minimum absolute path length between each pair of nodes, reflecting the global efficiency of parallel information transfer in the network [52],[74].
- The local efficiency of the *i*th node 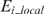 is defined as:
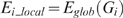
Since the *i*th node is not an element of the subgraph 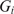, the local efficiency can be understood as a measure of the fault tolerance of the network, indicating how well each subgraph exchanges information when the index node is eliminated [52].

#### Evaluation of the small-world property

The concept of “small-world”, originally proposed by Watts and Strogatz [18], is strongly related to the average clustering coefficient, 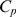, and the average shortest path length, 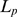, of the given graph. A real network would be considered as small-world if it meets the following criteria: 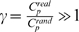 and 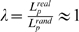 [18], in which 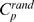 and 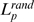 are the mean clustering coefficient and mean shortest path length of the random network. For the calculation of 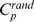 and 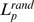, we followed the procedure which was used by Liu et al. in their recent study of disrupted small-world networks in schizophrenia [75]. In detail, we generated 100 random networks for each subject's anatomical network by a Markov-chain algorithm [12],[76],[77], in which the original connectivity matrix was randomly permuted with the same degree of distribution preserved. The permutation procedure was repeated until the topological structure of the original matrix was randomized [16]. Then we averaged across all 100 generated random networks to obtain the mean 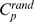 and 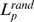. Small-world indices 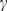 and 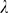 were then calculated for the binary and weighted anatomical networks of every individual.

#### Hubs and degree distribution

To further explore the configuration of the brain network, we examined the hub regions and degree distribution of the binary anatomical networks we constructed. Extended details can be found in Text S3.

### Statistical analysis

A two-sample *t-test* on the properties of binary and weighted networks was performed between the GI and HI groups using SPSS13.0, and a threshold value was set at 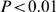 for significance. Please note that our database of healthy adults was divided into GI (70<FSIQ<120; 22 men and 20 women; age, 22.8±4.1 years) and HI (FSIQ> = 120; 22 men and 15 women; age, 24.9±3.3 years) groups according to their FSIQ scores in the same manner as in the previous study by our group [34], which was performed on the same dataset, for the sake of methodological consistency. We believe that an explanation for our choice of an FSIQ score of 120 as the cut-off value for general and high IQ groups division will be helpful for clarifying this study. In the Chinese Revised Wechsler Adult Intelligence Scale (WAIS-RC) we used, IQ classification in educational use is defined as: (1) Extremely Low (69 and below); Borderline (70–79); (3) Low Average (80–89); (4) Average (90–109); (5) High Average (110–119); (6) Superior (120–129); (7) Very Superior (130 and above). The IQ score of 120 is the cutting point which can be used to identify the subjects with “superior” and “very superior” intelligence. In addition, there are two previous studies which support this cutoff. In Waldmann's et al. [78] study, subjects between the ages of 18 and 30 were divided into groups based on their Satz-Mogel Wechsler Adult Intelligence Scale-Revised FSIQ scores: (a) Borderline (70 to 79); (b) Low Average (80 to 89); (c) Average (90 to 109); (d) High Average (110 to 119); (e) Superior (120 to 129). In another study by Karande et al. [79], ninety-five children with specific learning disabilities (aged 9–14 years) were divided into groups based on their nonverbal IQ scores obtained on the Wechsler Intelligence Scale for Children test: (i) average-nonverbal intelligence group (IQ 90–109), bright normal-nonverbal intelligence group (IQ 110–119), and (iii) superior-nonverbal intelligence group (IQ 120–129). In both studies, an IQ score of 120 was used as the cutoff for identifying the “superior group”. Because these two studies are basically comparable to our current study (although differing in populations, intelligence scale editions and IQ scores) they add credibility to the IQ cutoff in our investigation.

Partial correlations between intelligence test scores and global brain network properties (E, 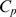, 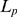, 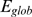) were performed across all subjects using SPSS 13.0, while controlling the effects of age and gender. The threshold value was set at 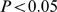 for significance. Furthermore, to localize the association with intellectual performance, partial correlations were also performed between the local efficiency (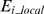) of each node region and the intelligence test scores across all subjects, while controlling for age and gender. The threshold value was set at 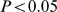 for significance (uncorrected).

### Methodological considerations

There are several methodological issues in our present study that need to be addressed.

First, a deterministic tractography method was utilized for network construction. We realize that this kind of fiber tracking method has a limited capacity for resolving crossing fiber bundles [71], which may lead to the loss of some existing fiber connections between brain regions or to the inclusion of some non-existent fibers. A probabilistic tractography method may be a better solution for future work as recent studies have demonstrated that this method is advantageous for overcoming the fiber crossing problem [27],[80],[81]. However, it is not applicable in our current investigation as only 12 diffusion directions were employed for data acquisition, an insufficient number for performing a valid probabilistic tracking method. However, the tensorline tracking method we used has been shown to be able to achieve robust and stable tracking results [68]–[71], which would help to increase the validity of our network construction.

Second, in contrast to a population-based network analysis, which may tend to exclude false-negative connections [32], our analysis of individual brain networks may lead to false-positive connections in each individual subject as a result of limitations that may arise from the image resolution and the tracking method. To increase the reliability of our work, we employed a threshold value on every individual brain network to exclude regional connections that have too few existing fiber bundles to be valid. Since the threshold value was carefully tested (see Table 2) and since consistent, stable results were obtained across all the different situations we tested, we believe that our investigation of individual brains was basically valid. However, more datasets using different populations should be tested in the future for further evaluation of our method.

Third, we developed our investigations from a binary to a weighted anatomical network by introducing different weighted indices. Although no existing studies can directly validate our method, our results showed that either using the number or the average FA value of the existing fiber bundles between two regions can lead to a network topology similar to that found in previous human brain network studies (see Text S2). The results of the statistical analyses indicated that using the number of fiber bundles that link two regions as an edge weight may be more appropriate than using the average FA when investigating the network properties associated with intellectual performance (see Text S2). We realize that the number of fiber bundles that we used here cannot represent the actual number of axonal fibers, but rather indicates the strength of the white matter connectivity between different brain regions. Although our findings provided relatively good support for this weight index, further examinations on other datasets are necessary.

Finally, a risk of this study is that some of the fiber tracts reconstructed by our method may not belong to the specific AAL region. This could happen if the white matter voxels included in the fiber tracking procedure were not truly adjacent to the cortex. Additionally, the choice of the relatively high FA threshold of 0.3 for the seed voxel in our current study might increase this possibility, since it may exclude low FA sub-cortical white matter areas as seed regions. To address this issue Gong et al. [32] removed white matter voxels from the unanalyzed AAL cortical mask if no cortical voxels existed within 2 mm^3^ of them. We believe that they have made an original and creative contribution to this issue. On the other hand, because no gold standard for identifying the nature of the removed white matter voxels exists, their method could lead to a risk of excluding fiber tracts that actually belong to the specific AAL region. This exclusion of potentially significant fiber tracts could subsequently affect the topological properties of the resulting brain anatomical network. Here, we would like to point out that the FA threshold of 0.3 we used in the current study was selected based on a somewhat similar study performed by Thottakara et al. [66], in which an FA threshold of 0.3 was used for selecting seed voxels to reconstruct fiber tracts originating from or terminating in different Brodmann areas utilizing the streamline tracking method. Although the details of our current study and theirs are not completely comparable, we believe that the FA threshold of 0.3 we used is basically valid, considering that the DTI images in our study were obtained from a 3.0-T MRI scanner using 12 non-collinear diffusion encoding directions, which are the same as those used in their study. Nevertheless, future investigations using a more sophisticated brain template will be necessary to better address this methodological limitation of our current study.

## Supporting Information

Text S1 Investigation of different brain parcellation schemes
(0.12 MB DOC)

Text S2 Investigation of different weighted indices
(0.06 MB DOC)

Text S3 Investigations of hubs and degree distribution in the brain network
(0.12 MB DOC)

Figure S1 Significant partial correlation between E and IQ scores under the scheme with 78 nodes.
(0.11 MB TIF)

Figure S2 Significant partial correlation between L_p_ and IQ scores under the scheme with 78 nodes.
(0.40 MB TIF)

Figure S3 Significant partial correlation between E__glob_ and IQ scores under the scheme with 78 nodes.
(0.39 MB TIF)

Figure S4 Significant partial correlation between L_p_ and IQ scores using average FA as weighted index.
(0.11 MB TIF)

Figure S5 Significant partial correlation between E__glob_ and IQ scores using average FA as weighted index.
(0.11 MB TIF)

Figure S6 Degree distributions of the group-based network and the binary networks of three randomly selected subjects.
(0.12 MB TIF)
