# Supplementary material for: Brain Anatomical Network and Intelligence
Source: PLoS Comput Biol. 2009 May 29;5(5):e1000395. doi: 10.1371/journal.pcbi.1000395 (PMC2683575; doi:10.1371/journal.pcbi.1000395)
Supplement: Text S1 — Investigation of different brain parcellation schemes (0.12 MB DOC) [file pcbi.1000395.s001.doc]

**Text S1. Investigation of different brain parcellation schemes**

**Introduction**

To investigate the impact of different brain parcellation scheme on the findings of our current study, we reconstructed the binary and weighted brain networks of all the 79 subjects according to a different brain division. The topological properties of the resultant networks were then explored as well as their association with intelligence test scores, which may help for providing convergent evidence to our findings.

**Methods and Results**

We employed the scheme of brain parcellation used by Gong et al. [1], in which the cerebral cortex of each subject was segmented into 78 regions (39 for each hemisphere with the subcortical structures excluded) using the AAL template [2]. Details about node definitions can be found in the main text. The definitions of each cortical or sub-cortical region of AAL template are listed in Table 1 with the following six subcortical structures excluded:

| Region name | Abbreviation |
| --- | --- |
| Hippocampus | HIP |
| Amygdala | AMYG |
| Caudate | CAU |
| Putamen | PUT |
| Pallidum | PAL |
| Thalamus | THA |

Binary and weighted brain anatomical networks were constructed on individual brains using the method we described in the main text under this parcellation scheme. A test for the threshold value used in network construction was performed and resulted in a value of 3 (see Table S1). Topological properties of the networks were calculated for small-world evaluation and statistical analyses were performed.

Both the binary and weighted anatomical networks of 78 node regions showed properties largely comparable to the scheme we used in the main text with 90 nodes across all subjects under the parcellation scheme, in which a small-world attribute was observed (see Table S2). These findings suggested that the modified method we used for anatomical network construction can lead to stable results for individual brains. Small-world properties may be a common finding in human brain networks.

As shown in Table S3, significant differences in network properties are observed between the GI and HI groups based on a parcellation of 78 brain regions. While controlling for age and gender, significant partial correlations between intelligence tests scores and topological properties of binary and weighted anatomical brain networks were found consistently in two different parcellation schemes (see Table S4, Figs.S1, S2 and S3).

**Short discussion**

The results of statistical analyses were basically comparable between the two different parcellation schemes, which may give support to the robustness of our methods. However, a parcellation scheme that used 90 nodes showed a much larger absolute value of the partial correlation coefficient and a much smaller *P*-value in the partial correlation analysis, as well as a smaller *P*-value from the two-sample *t-test* between the GI and HI groups. These differences might indicate that the subcortical structures (i.e. the thalamus) were important in human brain anatomical construction and may further affect the association between brain structural organization and intelligence.

Generally speaking, our findings showed good consistency between the two different brain segmentation schemes and further indicated the importance of subcortical structures in brain organization that can be associated with intelligence.

**References:**

1. Gong G, He Y, Concha L, Lebel C, Gross DW, et al. (2008) Mapping Anatomical Connectivity Patterns of Human Cerebral Cortex Using In Vivo Diffusion Tensor Imaging Tractography. Cereb Cortex.

2. Tzourio-Mazoyer N, Landeau B, Papathanassiou D, Crivello F, Etard O, et al. (2002) Automated anatomical labeling of activations in SPM using a macroscopic anatomical parcellation of the MNI MRI single-subject brain. Neuroimage 15: 273-289.

**Tables:**

Table S1. Test of the appropriate threshold value for the anatomical network construction on an individual brain under the brain parcellation scheme with 78 nodes.

| Threshold value | SOBCC, group mean (SD) |
| --- | --- |
| 1 | 78 (0.19) |
| 2 | 78 (0.47) |
| **3 *** | **78 (0.64) *** |
| 4 | 77 (0.80) |
| 5 | 77 (0.95) |

Abbreviations: SOBCC, Size of Biggest Connected Component.

Table S2. Topological properties including small-world indices of human brain networks in the current study using different brain parcellation schemes and different weighted indices.

| Brain networks | | | N | Cp | Lp | γ | λ | E_glob |
| --- | --- | --- | --- | --- | --- | --- | --- | --- |
| Binary network | Scheme 1 (90 nodes) | | 90 | 0.49 (±0.02) | 2.81 (±0.14) | 2.07 (±0.20) | 1.14 (±0.03) | 0.42 (±0.02) |
|  | Scheme 2 (78 nodes) | | 78 | 0.50 (±0.02) | 2.99 (±0.22) | 1.82 (±0.18) | 1.13(±0.03) | 0.41 (±0.02) |
| Weighted network | Scheme 1 (90 nodes) | Number of existing fibers | 90 | 0.55 (±0.02) | 0.15 (±0.03) | 2.20 (±0.21) | 1.27 (±0.08) | 11.04 (±2.10) |
|  |  | Average FA value | 90 | 0.49 (±0.02) | 6.49 (±0.44) | 2.08 (±0.20) | 1.13 (±0.02) | 0.18 (±0.01) |
|  | Scheme 2 (78 nodes) | Number of existing fibers | 78 | 0.56 (±0.02) | 0.15 (±0.03) | 1.98 (±0.20) | 1.29 (±0.12) | 12.10 (±2.53) |
|  |  | Average FA value | 78 | 0.51 (±0.02) | 7.22 (±0.69) | 1.82 (±0.18) | 1.14 (±0.03) | 0.17 (±0.01) |

Notes: N, Cp, Lp denote the number of nodes, average clustering coefficient and mean shortest path length of the network respectively. γ andλ denote the

small-world properties of the network. E_glob denotes the absolute global efficiency of the network. Detailed definitions can be found in the Materials and Methods

section of the main paper. The values from our current study are showed in the form of group mean (±SD), which were obtained by averaging across all the 79

subjects.

Table S3. Two-sample *t-tests* on the properties of binary and weighted networks between GI and HI groups using different brain parcellation schemes.

| Network Properties | | | Value, group mean (SD) | | *P-* value (Two-sample *t-test*)  (Equal variances assumed) |
| --- | --- | --- | --- | --- | --- |
|  |  |  | GI (n = 42) | HI (n = 37) | GI *vs* HI |
| E | 90 nodes | | 760.86(70.52) | 812.16(79.85) | 0.003 ***** |
|  | **78 nodes** | | **582.52(62.21)** | **625.54(77.50)** | **0.009 *** |
| Cp | Binary | 90 nodes | 0.48(0.01) | 0.49(0.02) | 0 .030 |
|  |  | **78 nodes** | **0.50(0.02)** | **0.50(0.02)** | **0 .716** |
|  | Weighted | 90 nodes | 0.54(0.02) | 0.55(0.02) | 0.014 |
|  |  | **78 nodes** | **0.56(0.02)** | **0.56(0.03)** | **0.315** |
| Lp | Binary | 90 nodes | 2.85(0.14) | 2.76(0.13) | 0.004 ***** |
|  |  | **78 nodes** | **3.05(0.20)** | **2.92(0.21)** | **0.006 *** |
|  | Weighted | 90 nodes | 0.16(0.02) | 0.14(0.02) | < 0.001 ***** |
|  |  | **78 nodes** | **0.15(0.03)** | **0.13(0.03)** | **0.002 *** |
| E_glob | Binary | 90 nodes | 0.42(0.02) | 0.43(0.02) | 0.003 ***** |
|  |  | **78 nodes** | **0.40(0.02)** | **0.42(0.02)** | **0.006 *** |
|  | Weighted | 90 nodes | 10.33(1.74) | 11.84(2.21) | 0 .001 ***** |
|  |  | **78 nodes** | **11.29(2.06)** | **13.01(2.72)** | **0 .002 *** |

The threshold value was set at $P<0.01$ with equal variances assumed.

Abbreviations: GI, General Intelligence; HI, High Intelligence.

Table S4. Partial correlation between topological properties and intelligence test scores across all subjects while controlling for age and gender using different brain parcellation schemes

| Topological properties | | | FSIQ | | PIQ | | VIQ | |
| --- | --- | --- | --- | --- | --- | --- | --- | --- |
|  |  |  | PCC | *P*-value | PCC | *P*-value | PCC | *P*-value |
| E | 90 nodes | | 0.242 | 0.034 ***** | 0.242 | 0.034 ***** | 0.213 | 0.063 |
|  | **78 nodes** | | **0.238** | **0.037 *** | **0.229** | **0.045 *** | **0.218** | **0.057** |
| Cp | Binary | 90 nodes | 0.138 | 0.231 | 0.117 | 0.311 | 0.142 | 0.218 |
|  |  | **78 nodes** | **-0.034** | **0.767** | **-0.037** | **0.752** | **-0.038** | **0.743** |
|  | Weighted | 90 nodes | 0.204 | 0.075 | 0.183 | 0.111 | 0.200 | 0.081 |
|  |  | **78 nodes** | **0.061** | **0.596** | **0.056** | **0.629** | **0.052** | **0.654** |
| Lp | Binary | 90 nodes | -0.262 | 0.021 ***** | -0.275 | 0.016 ***** | -0.221 | 0.053 |
|  |  | **78 nodes** | **-0.289** | **0.011 *** | **-0.282** | **0.013 *** | **-0.266** | **0.019 *** |
|  | Weighted | 90 nodes | -0.359 | 0.001 ***** | -0.342 | 0.002 ***** | -0.332 | 0.003 ***** |
|  |  | **78 nodes** | **-0.319** | **0.005 *** | **-0.302** | **0.008 *** | **-0.295** | **0.009 *** |
| E_glob | Binary | 90 nodes | 0.264 | 0.021 ***** | 0.272 | 0.017 ***** | 0.227 | 0.047 ***** |
|  |  | **78 nodes** | **0.281** | **0.013 *** | **0.268** | **0.019 *** | **0.263** | **0.021 *** |
|  | Weighted | 90 nodes | 0.308 | 0.006 ***** | 0.281 | 0.013 ***** | 0.296 | 0.009 ***** |
|  |  | **78 nodes** | **0.289** | **0.011 *** | **0.263** | **0.021 *** | **0.276** | **0.015 *** |

The threshold value was set at *P < 0.05* for significance.

Abbreviations: FSIQ, Full Scale IQ; PIQ, performance IQ; VIQ, verbal IQ; PCC, partial correlation coefficient.
