# Supplementary material for: Brain Anatomical Network and Intelligence
Source: PLoS Comput Biol. 2009 May 29;5(5):e1000395. doi: 10.1371/journal.pcbi.1000395 (PMC2683575; doi:10.1371/journal.pcbi.1000395)
Supplement: Text S2 — Investigation of different weighted indices (0.06 MB DOC) [file pcbi.1000395.s002.doc]

**Text S2. Investigation of different weighted indices**

**Introduction**

It remains as an open question about how to weight the brain network properly. To provide more complete picture to our current study, we investigated the topological properties of brain network constructed using different weighted index other than the number of existing fiber bundles between two brain regions, and further explored the association between intelligence test scores and the resultant networks across all subjects.

**Methods and Results**

In addition to the number of existing fiber bundles between two connecting regions, we employed the average FA value of all the reconstructed fiber bundles between two brain regions as a weighted index. Topological properties of the resulting weighted anatomical network were calculated for small-world evaluation. Subsequent statistical analyses included a two sample *t-test* between the GI and HI groups and a partial correlation analyses between IQ scores and brain network properties across all subjects.

As showed by Table S2 in Text S1, although the network topological properties were different between weighted networks using different indices, a prominent small-world attribute was observed when the network was weighted by average FA value of the existing fiber bundles between brain regions. Our result suggested that the small-world property is a common finding in the human brain. Significant differences in network properties were observed between the GI and HI groups (Table S5). As showed in Table S6, significant correlations between network properties and intelligence tests scores were found in the anatomical networks that used an average FA value of the connecting fiber bundles as weighted index (Figs.S4, S5). Please note that the results of inter-groups comparisons and partial correlation analyses performed using the average FA value as weighted index were observed under the threshold value of 3 for network construction while the brain parcellation scheme of 90 nodes were employed.

**Short discussion**

The results of statistical analyses between networks using two different weighted indices were largely comparable, giving support to the validation of our network construction method. However, in all cases, weighted networks using the number of existing fiber bundles showed a much larger absolute value of the partial correlation coefficient and a much smaller *P*-value in the partial correlation analysis, as well as a much smaller *P*-value in the two-sample *t-test*. This suggests that the average FA value of the connecting fiber bundles between two brain regions may be not a very appropriate index in the investigation associated with intelligence in our current study. This may be due to the various kinds of topological patterns of the connecting fiber bundles, and the average across them may be too coarse for describing the edge weight. On the other hand, the existing number of fiber bundles connecting two brain regions may represent the strength of white matter connectivity and reflect the potential ability of parallel information processing and may be able to provide a better description of the anatomical network.

**Tables:**

Table S5. Two-sample *t-test* on topological properties of networks constructed using different weighted indices between GI and HI groups.

| Network Properties | | Value, group mean (SD) | | *P-* value (Two-sample *t-test*)  (Equal variances assumed) |
| --- | --- | --- | --- | --- |
|  |  | GI (n = 42) | HI (n = 37) | GI *vs* HI |
| Cp | FiberNum | 0.54(0.02) | 0.55(0.02) | 0.014 |
|  | **FiberFA** | **0.49(0.15)** | **0.50(0.02)** | **0.025** |
| Lp | FiberNum | 0.16(0.02) | 0.14(0.02) | < 0.001 ***** |
|  | **FiberFA** | **6.62(0.44)** | **6.34(0.41)** | **0.004 *** |
| E_glob | FiberNum | 10.33(1.74) | 11.84(2.21) | 0 .001 ***** |
|  | **FiberFA** | **0.18(0.01)** | **0.19(0.01)** | **0 .005 *** |

The threshold value was set at $P<0.01$ with equal variances assumed.

Abbreviations: GI, General Intelligence; HI, High Intelligence.

Table S6. Partial correlation between topological properties of networks constructed using different weighted indices and intelligence test scores across all subjects while controlling for age and gender.

| Topological properties | | FSIQ | | PIQ | | VIQ | |
| --- | --- | --- | --- | --- | --- | --- | --- |
|  |  | PCC | *P*-value | PCC | *P*-value | PCC | *P*-value |
| Cp | FiberNum | 0.204 | 0.075 | 0.183 | 0.111 | 0.200 | 0.081 |
|  | **FiberFA** | **0.153** | **0.184** | **0.131** | **0.256** | **0.158** | **0.171** |
| Lp | FiberNum | -0.359 | 0.001 ***** | -0.342 | 0.002 ***** | -0.332 | 0.003 ***** |
|  | **FiberFA** | **-0.236** | **0.039 *** | **-0.211** | **0.066** | **-0.239** | **0.036 *** |
| E_glob | FiberNum | 0.308 | 0.006 ***** | 0.281 | 0.013 ***** | 0.296 | 0.009 ***** |
|  | **FiberFA** | **0.225** | **0.049 *** | **0.184** | **0.109** | **0.243** | **0.033 *** |

The threshold value was set at *P < 0.05* for significance.

Abbreviations: FSIQ, general IQ; PIQ, performance IQ; VIQ, verbal IQ; PCC, partial correlation coefficient.
