# Supplementary material for: Brain Anatomical Network and Intelligence
Source: PLoS Comput Biol. 2009 May 29;5(5):e1000395. doi: 10.1371/journal.pcbi.1000395 (PMC2683575; doi:10.1371/journal.pcbi.1000395)
Supplement: Text S3 — Investigations of hubs and degree distribution in the brain network (0.12 MB DOC) [file pcbi.1000395.s003.doc]

**Text S3: Investigations of hubs and degree distribution in the brain network**

**Introduction**

To further explore the configuration of the brain network, we performed an examination of the hub regions and degree distribution on the group-based network generated by averaging across the binary networks of all 79 subjects (showed in Fig.2 in the main text). We also performed our investigation of hubs and degree distribution on the binary networks of three randomly selected subjects to achieve a more complete picture. Please note that the results of the following examinations were observed at a threshold value of 3 for network construction.

**Methods and Results**

To generate the group-based network, a value of 0.5 was used as a threshold for the mean map which was obtained by averaging across the binary connectivity matrixes of all 79 subjects, as showed in Figure 2. This threshold value means that only connectivity which occurs between corresponding pairs of brain regions in more than half of the subjects was included in the group-based network and thus may capture the underlying anatomical connectivity pattern across all the subjects.

*Examination of hub regions*

The hubs of the network were determined by the node degree (defined in the main text), in which a node region was identified as the hub of the network if its node degree was at least one standard deviation greater than the average node degree of the whole network [1]. In the present study, we found hubs, most of which had also been identified by some previous studies (see Table S7).

*Examination of degree distribution*

Previous studies have suggested that networks with small-world attributes can be divided into different categories according to their node degree distribution [2]. Our examinations in the group-based network as well as in the binary networks of three randomly selected subjects indicated that the node degree distribution we observed was somewhat heavy-tailed (see the left column of Fig.S6). As suggested by previous studies, three possible forms of distribution may be fitted to the frequency of node degree: the power-law, $\text{ p(k) \textasciitilde k}^{\alpha\text{-1}}$; the exponential, $\text{ p(k) \textasciitilde e}^{\text{-}\alpha\text{k}}$ and the exponentially truncated power-law, $\text{ p(k) \textasciitilde k}^{\alpha\text{-1}}\text{e}^{\text{k/k}_{\text{c}}}$. Here a cumulative distribution was introduced in order to reduce the effects of noise on the relatively small data set [1,3,4], and the goodness of fit was evaluated by $\text{R}^{\text{2 }}$values[[1]](#footnote-2) [1,5]. Of the three distributions, the exponentially truncated power law was found to be the best-fit model (see the right column of Fig.S6 as well as Table S8). Our results were consistent with those reported by previous studies of functional brain networks [3], as well as of anatomical brain networks [1,5-8].

**Short discussion**

Based on the node degree, 15 hub regions were identified in the brain network we constructed (see Table S7). We noticed that most of the hub regions we found had previously been identified as hub regions in studies of human brain anatomical [1,5,7], morphological [6,8], or functional networks [3]. Notably, the PCUN was observed as a hub region in various kinds of human brain networks across different studies, and its approximately equivalent region (i.e., area 7) has also been reported as pivotal nodes in the cortical network of macaques [9,10]. Previous studies exploring the neuroanatomy of cognitive functions have demonstrated the important roles of the PCUN in multiple highly integrated functional systems. For example, the PCUN has been involved in dealing with visuospatial imagery, self-processing, episodic memory retrieval, and consciousness [11]. Moreover, our results indicated that the thalamic region may be an important hub region although it has seldom been reported before.

In the present study, we also found that the node degree distribution of the brain anatomical network we constructed was best fitted by the exponentially truncated power-law equation. This finding indicates that the brain network may include some pivotal nodes (i.e., hubs) but prevents the existence of huge hubs with too much load. Our results are consistent with the findings of previous studies [1,3,6,7,12].

**References:**

1. Gong G, He Y, Concha L, Lebel C, Gross DW, et al. (2009) Mapping anatomical connectivity patterns of human cerebral cortex using in vivo diffusion tensor imaging tractography. Cereb Cortex 19: 524-536.

2. Amaral LA, Scala A, Barthelemy M, Stanley HE (2000) Classes of small-world networks. Proc Natl Acad Sci U S A 97: 11149-11152.

3. Achard S, Salvador R, Whitcher B, Suckling J, Bullmore E (2006) A resilient, low-frequency, small-world human brain functional network with highly connected association cortical hubs. J Neurosci 26: 63-72.

4. Strogatz SH (2001) Exploring complex networks. Nature 410: 268-276.

5. Iturria-Medina Y, Sotero RC, Canales-Rodriguez EJ, Aleman-Gomez Y, Melie-Garcia L (2008) Studying the human brain anatomical network via diffusion-weighted MRI and Graph Theory. Neuroimage 40: 1064-1076.

6. He Y, Chen ZJ, Evans AC (2007) Small-world anatomical networks in the human brain revealed by cortical thickness from MRI. Cereb Cortex 17: 2407-2419.

7. Hagmann P, Cammoun L, Gigandet X, Meuli R, Honey CJ, et al. (2008) Mapping the structural core of human cerebral cortex. PLoS Biol 6: e159.

8. Chen ZJ, He Y, Rosa-Neto P, Germann J, Evans AC (2008) Revealing modular architecture of human brain structural networks by using cortical thickness from MRI. Cereb Cortex 18: 2374-2381.

9. Honey CJ, Kotter R, Breakspear M, Sporns O (2007) Network structure of cerebral cortex shapes functional connectivity on multiple time scales. Proc Natl Acad Sci U S A 104: 10240-10245.

10. Sporns O, Honey CJ, Kotter R (2007) Identification and classification of hubs in brain networks. PLoS ONE 2: e1049.

11. Cavanna AE, Trimble MR (2006) The precuneus: a review of its functional anatomy and behavioural correlates. Brain 129: 564-583.

12. Achard S, Bullmore E (2007) Efficiency and cost of economical brain functional networks. PLoS Comput Biol 3: e17.

**Tables:**

Table S7. Brain regions identified as hubs.

| Regions | Node degree | Identified as a hub in different networks | Identified as a hub in previous human brain networks studies |
| --- | --- | --- | --- |
| PCUN_L | 21 | G-S_1_2_3 | AN^[1], [5], [7]^ and FN^[3]^ |
| PCUN_R | 18 | G-S_1_2_3 | AN^[1], [5], [7]^ and FN^[3]^ |
| INS_L | 17 | G-S_1_2_3 | AN^[5]^ |
| MOG_L | 14 | G-S_2_3 | AN^[1]^ and FN^[3]^ |
| PUT_R | 14 | G-S_1_2_3 | AN^[5]^ |
| SFG_R | 13 | G-S_3 | AN^[5], [6], [8]^ and FN^[3]^ |
| PCC_R | 13 | G | AN^[7]^ |
| SPG_R | 13 | G-S_3 | AN^[6], [8]^ and FN^[3]^ |
| CAL_R | 12 | G | AN^[7]^ and FN^[3]^ |
| THA_L | 12 | G-S_2_3 | -- |
| SFG_L | 11 | G-S_1_3 | AN^[5], [6], [8]^ and FN^[3]^ |
| MCG_R | 11 | G-S_2 | FN^[3]^ |
| PoCG_R | 11 | G-S_3 | AN^[6]^ and FN^[3]^ |
| PUT_L | 11 | G-S_1_2_3 | AN^[5]^ |
| MTG_L | 11 | G-S_1_2_3 | AN^[6], [8]^ and FN^[3]^ |

Note: “L” indicates that the region was located in the left hemisphere, and “R” stands for the right hemisphere. “G” stands for group-based network and “S” stands for subjects (e.g. G-S_1_2_3 represents that the brain region was identified as hub in the group-based network as well as in subjects 1, 2 and 3).

[1], [3], [5], [6], [7], [8]: represent the corresponding references. “--" stands for “Not reported” in these earlier studies.

Abbreviations: The abbreviations of brain regions were defined in Table 1 of the main text. AN, Anatomical Networks; FN, Functional Networks.

Table S8. $\text{R}^{\text{2 }}$values of three types of fitted curves in the group-based brain network and the binary brain networks of three randomly selected subjects.

| Brain network | Pl $\text{ p(k) \textasciitilde k}^{\alpha\text{-1}}$ | Exp $\text{ p(k) \textasciitilde e}^{\text{-}\alpha\text{k}}$ | Etp $\text{ p(k) \textasciitilde k}^{\alpha\text{-1}}\text{e}^{\text{k/k}_{\text{c}}}$ |
| --- | --- | --- | --- |
| Group | 0.6773 | 0.9213 | **0.9877** |
| Subject 1 | 0.5655 | 0.8148 | **0.8681** |
| Subject 2 | 0.6261 | 0.8971 | **0.9705** |
| Subject 3 | 0.7358 | 0.9134 | **0.9598** |

Abbreviations: Pl, Power-law; Exp, Exponential; Etp, Exponentially truncated power-law.

1. R^2^, or called Coefficient of Determination, is a statistical measure that represents how well the fit curve is. It is defined as , where , is the sum squared error of the regressed curve, , is the total of sum of squares of the fitted data, here, *Y* is the regression curve, and *X* is the real data. Therefore, an R^2^ close to 1 indicates a better fit. [↑](#footnote-ref-2)
