# Supplementary figures and images for: Brain Anatomical Network and Intelligence
Source: PLoS Comput Biol. 2009 May 29;5(5):e1000395. doi: 10.1371/journal.pcbi.1000395 (PMC2683575; doi:10.1371/journal.pcbi.1000395)

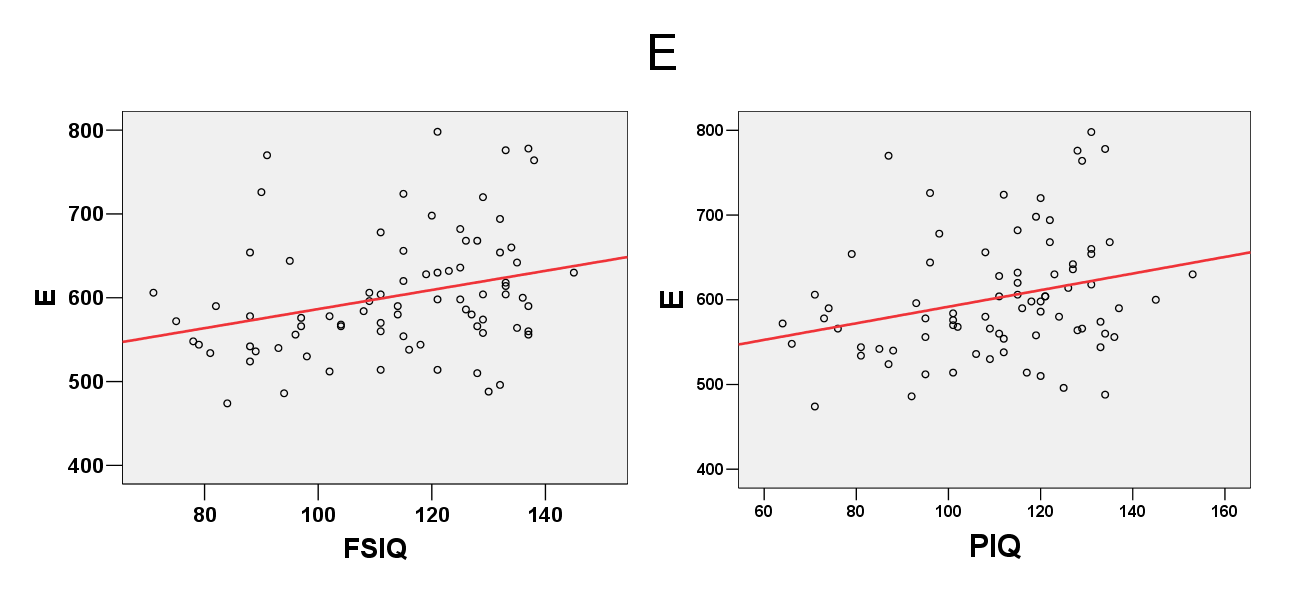

Supplement: Figure S1 — Significant partial correlation between E and IQ scores under the scheme with 78 nodes. (0.11 MB TIF) [file pcbi.1000395.s004.tif]

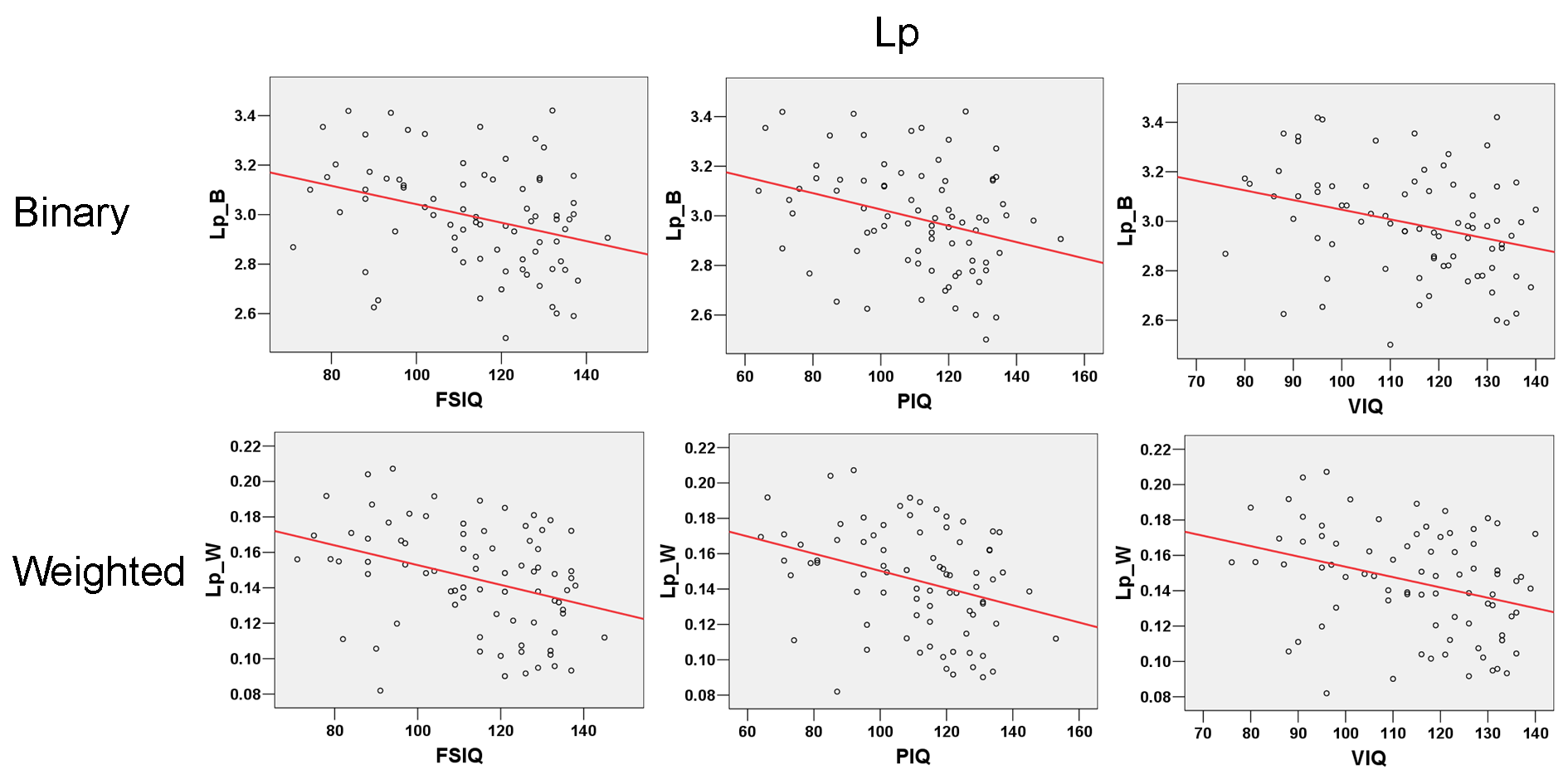

Supplement: Figure S2 — Significant partial correlation between Lp and IQ scores under the scheme with 78 nodes. (0.40 MB TIF) [file pcbi.1000395.s005.tif]

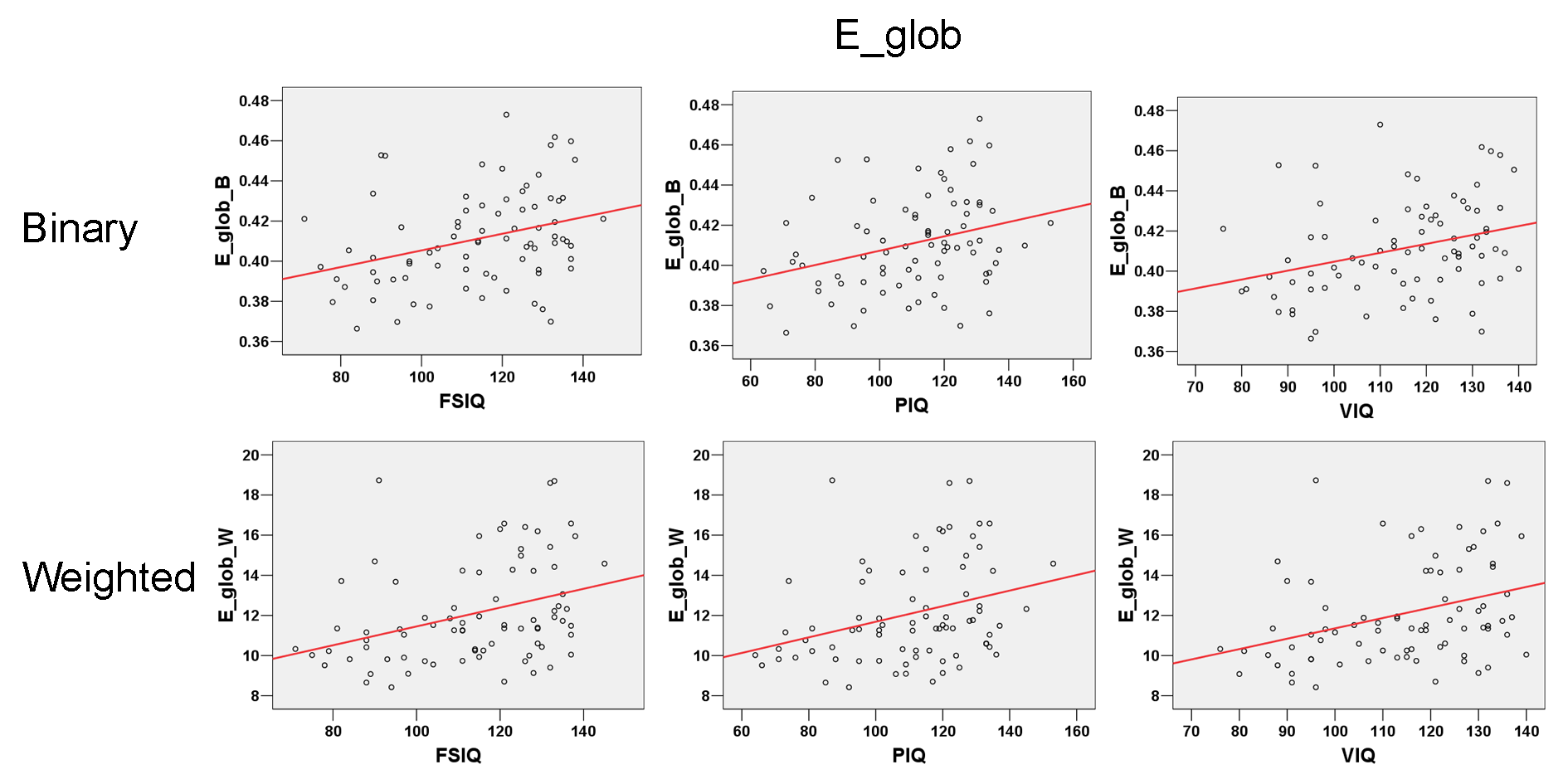

Supplement: Figure S3 — Significant partial correlation between E_glob and IQ scores under the scheme with 78 nodes. (0.39 MB TIF) [file pcbi.1000395.s006.tif]

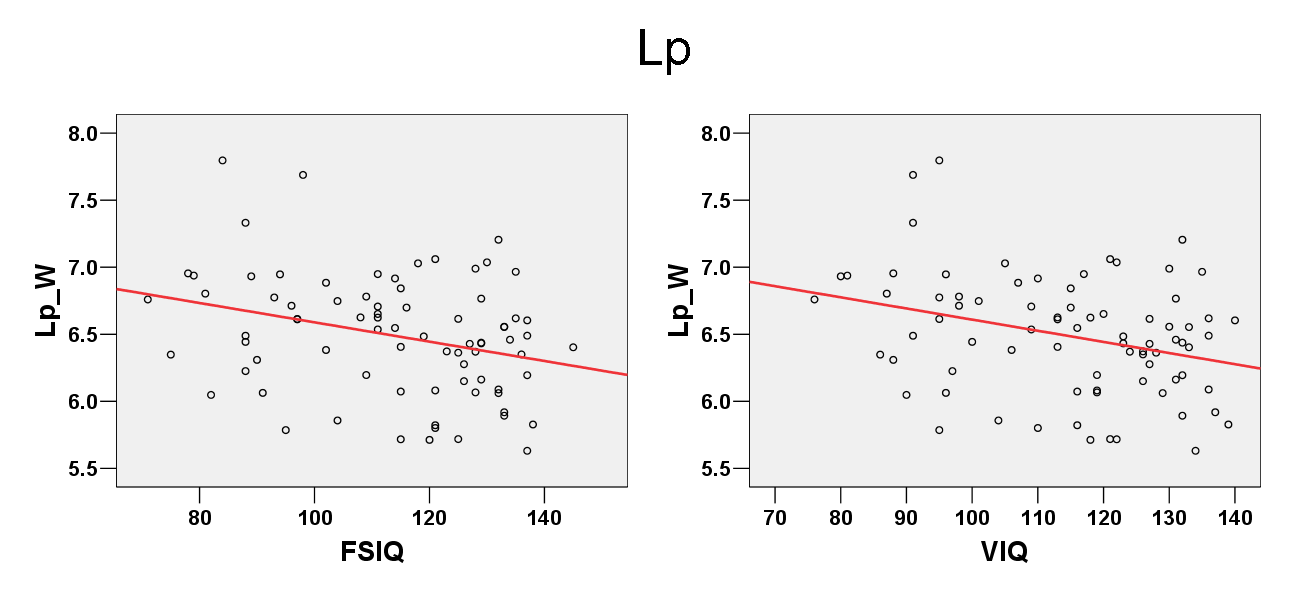

Supplement: Figure S4 — Significant partial correlation between Lp and IQ scores using average FA as weighted index. (0.11 MB TIF) [file pcbi.1000395.s007.tif]

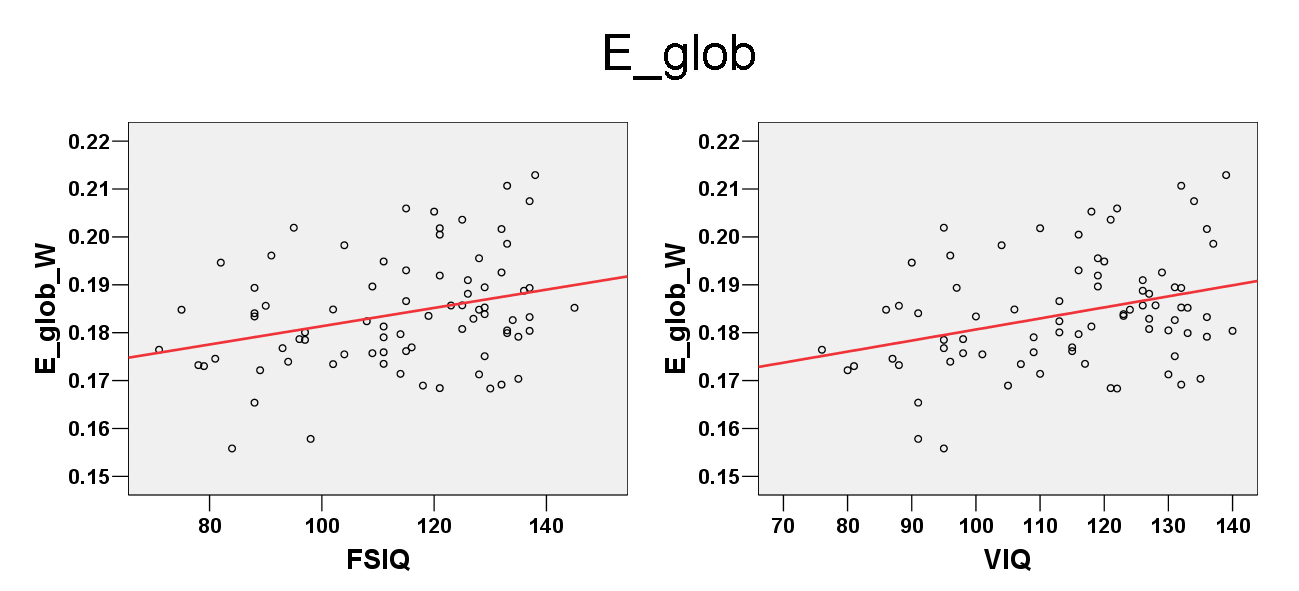

Supplement: Figure S5 — Significant partial correlation between E_glob and IQ scores using average FA as weighted index. (0.11 MB TIF) [file pcbi.1000395.s008.tif]

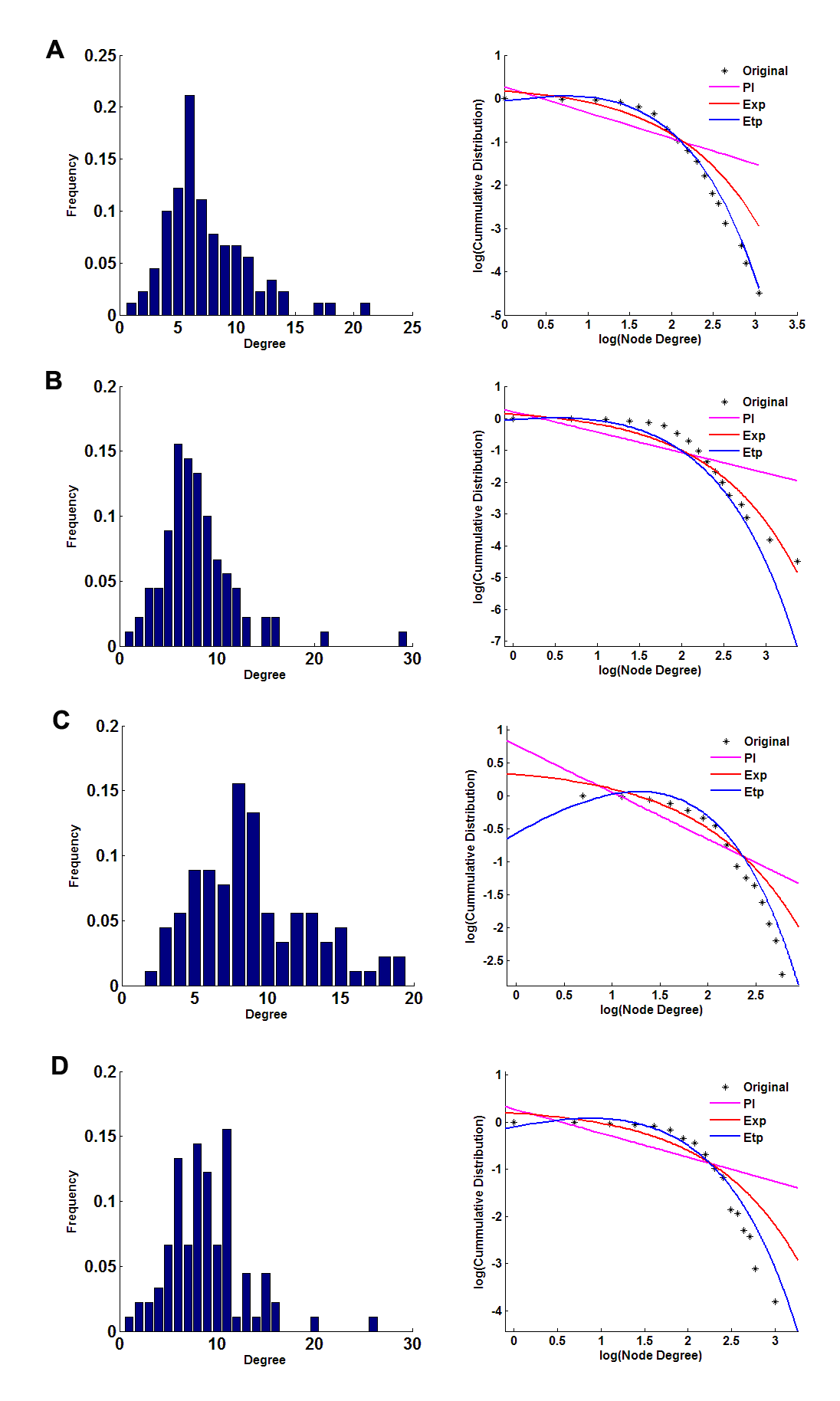

Supplement: Figure S6 — Degree distributions of the group-based network and the binary networks of three randomly selected subjects. (0.12 MB TIF) [file pcbi.1000395.s009.tif]
